# β-Cyclodextrin- and adamantyl-substituted poly(acrylate) self-assembling aqueous networks designed for controlled complexation and release of small molecules

**DOI:** 10.3762/bjoc.13.183

**Published:** 2017-09-07

**Authors:** Liang Yan, Duc-Truc Pham, Philip Clements, Stephen F Lincoln, Jie Wang, Xuhong Guo, Christopher J Easton

**Affiliations:** 1 Department of Chemistry, University of Adelaide, Adelaide, SA 5005, Australia; 2 State Key Laboratory of Chemical Engineering, East China University of Science and Technology, Shanghai 200237, China; 3 Research School of Chemistry, Australian National University, Canberra, ACT 0200, Australia

**Keywords:** controlled release, cyclodextrin, network, poly(acrylate), self-assembly

## Abstract

Three aqueous self-assembling poly(acrylate) networks have been designed to gain insight into the factors controlling the complexation and release of small molecules within them. These networks are formed between 8.8% 6^A^-(2-aminoethyl)amino-6^A^-deoxy-6^A^-β-cyclodextrin, β-CDen, randomly substituted poly(acrylate), PAAβ-CDen, and one of the 3.3% 1-(2-aminoethyl)amidoadamantyl, ADen, 3.0% 1-(6-aminohexyl)amidoadamantyl, ADhn, or 2.9% 1-(12-aminododecyl)amidoadamantyl, ADddn, randomly substituted poly(acrylate)s, PAAADen, PAAADhn and PAAADddn, respectively, such that the ratio of β-CDen to adamantyl substituents is ca. 3:1. The variation of the characteristics of the complexation of the dyes methyl red, methyl orange and ethyl orange in these three networks and by β-cyclodextrin, β-CD, and PAAβ-CDen alone provides insight into the factors affecting dye complexation. The rates of release of the dyes through a dialysis membrane from the three aqueous networks show a high dependence on host–guest complexation between the β-CDen substituents and the dyes as well as the structure and the viscosity of the network as shown by ITC, ^1^H NMR and UV–vis spectroscopy, and rheological studies. Such networks potentially form a basis for the design of controlled drug release systems.

## Introduction

The formation of self-assembling aqueous polymer networks through the complexation of hydrophobic polymer substituents by cyclodextrin oligomers [1–4] and cyclodextrin substituted polymers [5–19] to form cross-links between polymer strands is well-established. Depending upon their composition, these networks and related systems retain drug and similar molecules to varying extents which renders them of interest as potential drug delivery systems [20–47]. Generally, the retention and the release of the drug is controlled by the thermodynamics of drug complexation and in some systems the drug release is stimulated by either pH variation [28,30,34] or light irradiation [38,45]. The drug types include small molecular species, exemplified by diflunisal, fluconazole [40] and curcumin [37], along with larger species exemplified by RNA and DNA segments [26,32–33,36,39,47]. Some systems are designed to target specific tissues [26,35].

**Figure 1:**
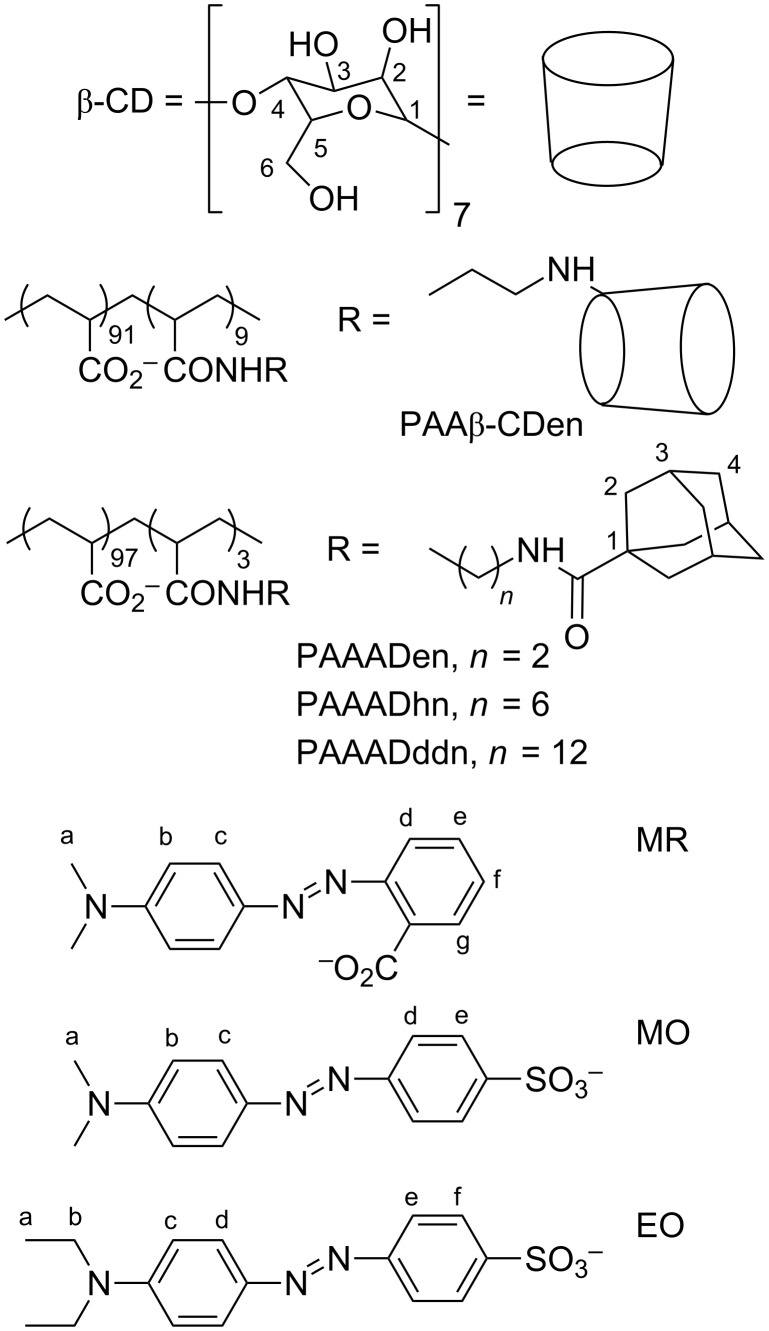
β-Cyclodextrin- and adamantyl-substituted poly(acrylate)s PAAβ-CDen, PAAADen, PAAADhn, and PAAADddn (carbon atoms labelled numerically), and the anionic dyes methyl red, MR, methyl orange, MO, and ethyl orange, EO (carbon atoms labelled alphabetically).

We are particularly interested in the extent to which small molecule guest complexation and release characteristics may be designed into the structure of aqueous networks formed between a β-cyclodextrin-substituted poly(acrylate) and three adamantyl-substituted poly(acrylate)s. Accordingly, we report an ITC, ^1^H NMR and UV–vis spectroscopic and rheological study of three self-assembling networks formed between the 8.8% 6^A^-(2-aminoethyl)amino-6^A^-deoxy-6^A^-β-cyclodextrin, β-CDen, randomly substituted poly(acrylate), PAAβ-CDen [13], and 3.3% 1-(2-aminoethyl)amidoadamantyl, ADen, 3.0% 1-(6-aminohexyl)amidoadamantyl, ADhn, or 2.9% 1-(12-aminododecyl)amidoadamantyl, ADddn, randomly substituted poly(acrylate)s, PAAADen [11], PAAADhn [15] and PAAADddn [15], respectively, where the poly(acrylate) is of 250 kDa average molecular weight prior to substitution (Figure 1). The network formation is driven by the β-CDen substituents complexing the adamantyl substituents, ADen, ADhn or ADddn, to form cross-links between the PAAβ-CDen strands and the PAAADen, PAAADhn or PAAADddn strands. The adamantyl group is selected as the guest substituent as it is strongly complexed by β-CD [48], β-CD oligomers [3–4] and β-CD-substituted polymers [10,14,16], and drives the self-assembly of aqueous chitosan [1,5,9], hyaluronic acid [8–9] and poly(acrylate) networks [14–15,19]. In aqueous solutions equimolar in PAAβ-CDen strands and PAAADen, PAAADhn or PAAADddn strands, the concentration of the β-CDen substituents is in ca*.* three-fold excess over that of the adamantyl substituents as a consequence of the ca*.* three-fold greater percentage substitution of PAAβ-CDen. Thus, when host–guest complexation between the poly(acrylate) substituents of the network is complete, ca*.* two thirds of the β-CDen substituents remain available to complex other hydrophobic species exemplified by the dyes methyl red, MR, methyl orange, MO, and ethyl orange, EO, chosen for this study (Figure 1).

## Results and Discussion

### Isothermal titration calorimetry (ITC) studies of substituted poly(acrylate) network formation

**Figure 2:**
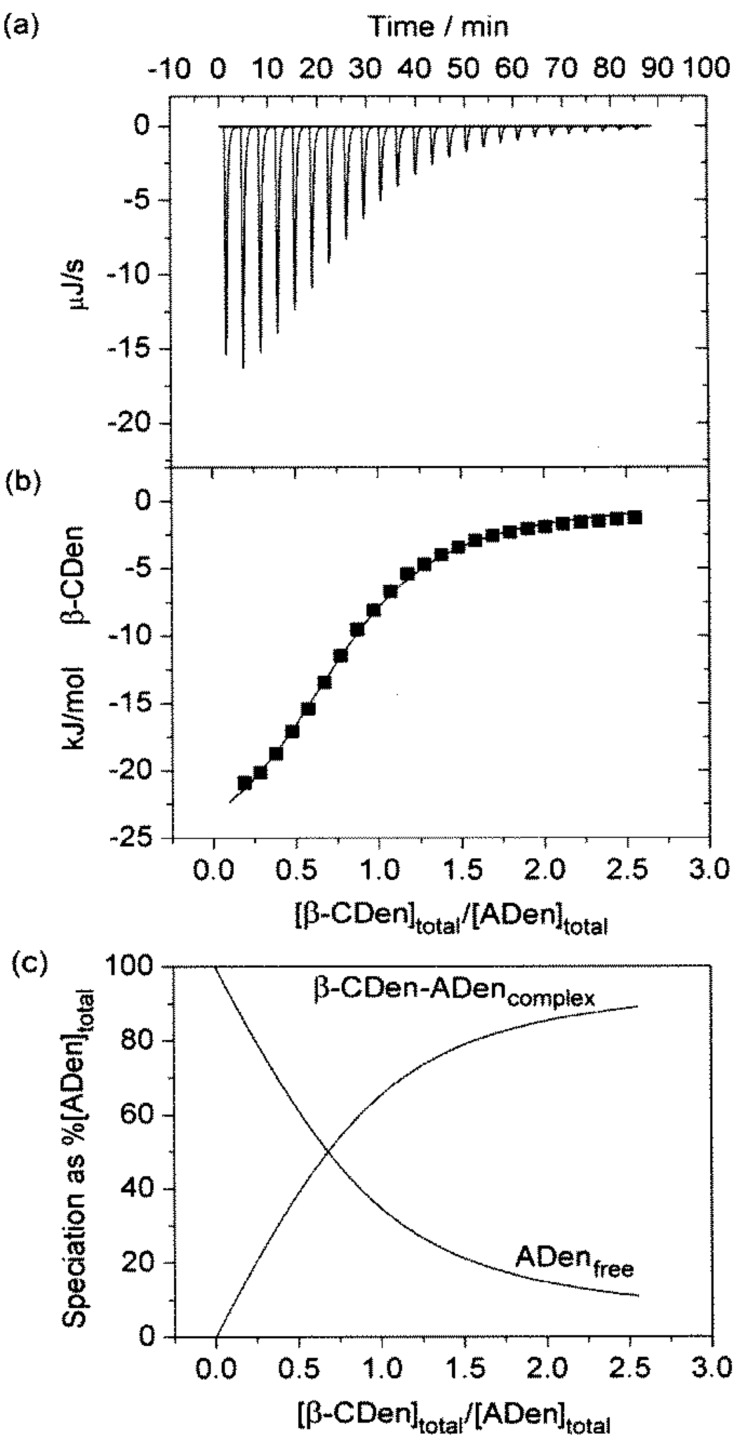
ITC data for the PAAβ-CDen/PAAADen, system obtained in aqueous Na_2_HPO_4_/KH_2_PO_4_ pH 7.0 buffer at *I* = 0.10 mol dm^−3^. (a) Titration of 10 mm^3^ aliquots of 0.62 wt % PAAβ-CDen ([β-CDen] = 2.84 × 10^−3^ mol dm^−3^) into 1.46 cm^3^ of 0.062 wt % PAAADen ([ADen] = 2.06 × 10^−4^ mol dm^−3^). (b) The solid curve shows the best fit of an algorithm for host–guest complexation between the β-CDen and ADen substituents to the titration data points. (c) Speciation plots showing for the variation of the [β-CDen–ADen]_complex_ and of the [ADen]_free_ as percentage a of [ADen]_total_ = 100%.

**Figure 3:**
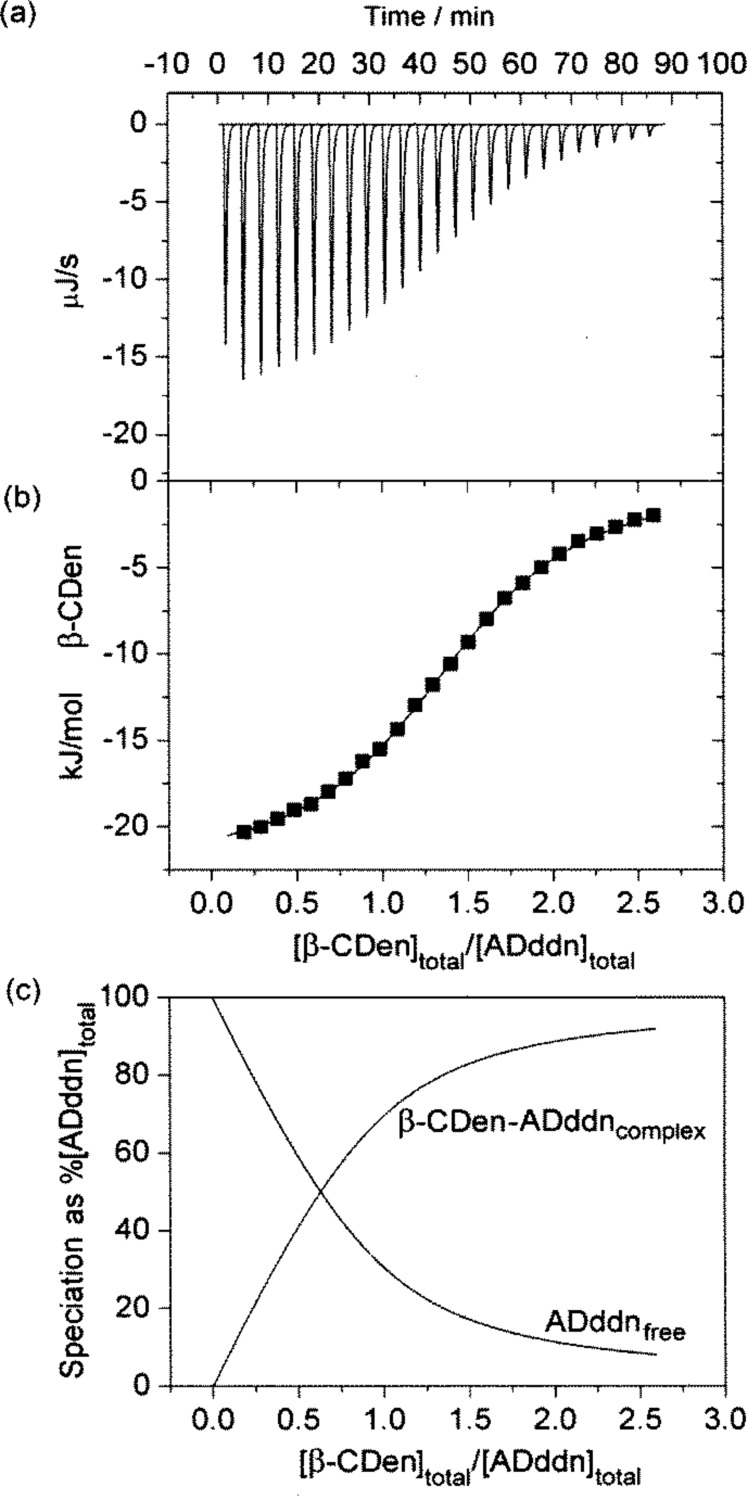
ITC data for the PAAβ-CDen/PAAADddn system obtained in aqueous Na_2_HPO_4_/KH_2_PO_4_ pH 7.0 buffer at *I* = 0.10 mol dm^−3^. (a) Titration data: for each system 10 mm^3^ aliquots of a 0.62 wt % PAAβ-CDen solution ([β-CDen] = 2.84 × 10^−3^ mol dm^−3^) into 1.46 cm^3^ of 0.064 wt % PAAADddn ([ADddn] = 1.91 × 10^−4^ mol dm^−3^). (b) The solid curve shows the best fit of an algorithm for host–guest complexation between the β-CDen and ADddn substituents to the titration data. (c) Speciation plot showing the variation of [β-CDen–ADddn]_complex_ and of [ADen]_free_ as a percentage of [ADddn]_total_ = 100%.

In aqueous solution, the host β-CDen substituents of PAAβ-CDen complex the guest adamantyl substituents of PAAADen, PAAADhn and PAAADddn to form poly(acrylate) networks as exemplified by the PAAβ-CDen/PAAADen system according to Equation 1. The complexation constant, *K*, for the host–guest complexation between the β-CDen and ADen substituents within the network is given by Equation 2. The data for the titration of a PAAβ-CDen solution into a PAAADen, a PAAADhn, or a PAAADddn solution together with the best-fit of an algorithm for a single complexation (Equation 1) for the PAAβ-CDen/PAAADen system (and analogous equations for the other two systems) to these data appear in Figure 2 and Figure 3 and Figure S1 in Supporting Information File 1. The derived *K* and the corresponding Δ*H*, *T*Δ*S*, and *N* values are given in Table 1. The ratio of the number of ADen substituents of PAAADen complexed by a single β-CDen substituent of PAAβ-CDen, *N*, is expected to be unity for unhindered 1:1 complexation and corresponds to the ratio of complexed β-CDen substituents to complexed adamantyl substituents at the inflexion point of the ITC titration curve (Equation 3). However, the *N =* 0.78 and 0. 87 derived for each β-CDen substituent complexing either a ADen or ADhn substituent, respectively, are less than unity as also observed in other studies [1,3–5,8]. This is attributed to either steric hindrance by the poly(acrylate) backbone, or hydrophobic association of the adamantyl substituents, or both, hindering complexation.

[1]
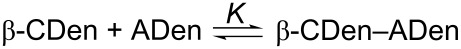


[2]
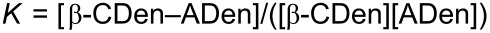


[3]
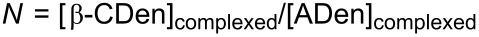


**Figure 4:**
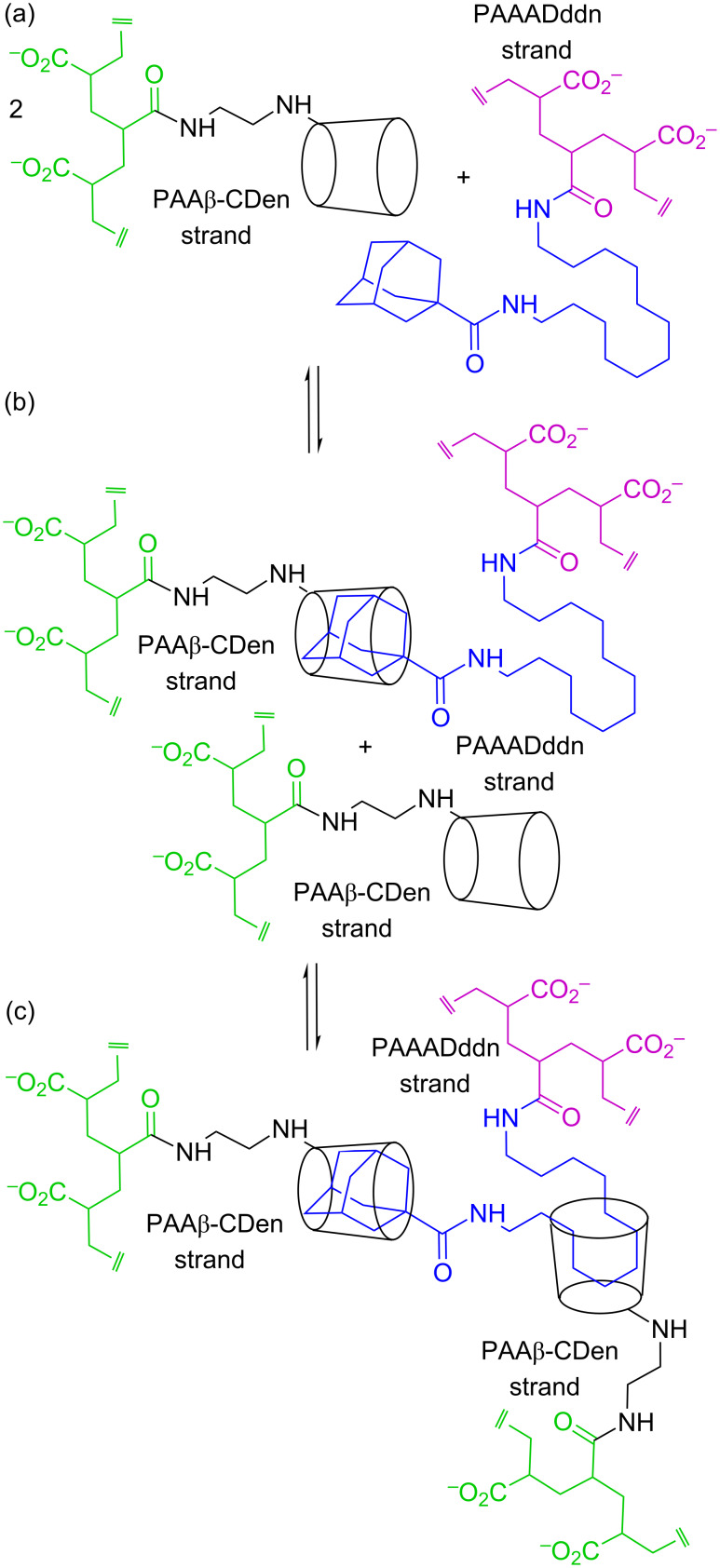
Representation of ditopic complexation of an ADddn substituent of PAAADddn, by two β-CDen substituents of PAAβ-CDen through initial complexation of the adamantyl group followed by complexation of the dodecyl linker in the sequence (a) to (b) to (c).

The PAAβ-CDen/PAAADddn system contrasts with the other two systems in that *N* = 1.45 is consistent with one β-CDen substituent complexing the adamantyl group of the ADddn substituent and a second β-CDen substituent complexing its dodecyl tether in the sequence shown in Figure 4. (Alternatively, the dodecyl tether may be complexed first followed by complexation of the adamantyl group. A further possibility is that the adamantyl group may pass through the annulus of one β-CDen substituent to be complexed by a second β-CDen substituent.) The titration curve (Figure 3) could not be resolved into two separate curves consistent with the two complexations being akin to a chelation process which arises from the greater length and flexibility of the dodecyl tether. Ideally, *N* = 2 should be observed for such a process, but it appears that either the steric hindrance or hydrophobic association of the adamantyl substituents, or both, restrict *N* to <2. The 2D NOESY ^1^H NMR spectrum of the PAAβ-CDen/PAAADddn system confirms the complexation of both the adamantyl group and its dodecyl tether as is discussed below.

**Table 1:** Parameters for host–guest complexation between PAAβ-CDen and PAAADen, PAAADhn, or PAAADddn.^a^

Host	PAAβ-CDen^b^		

Guest	PAAADen	PAAADhn	PAAADddn

10^−3^*K* dm^3^ mol^−1^	28.2 ± 0.15	28.4 ± 0.15	39.5 ± 0.08
Δ*H* kJ mol^−1^	−27.81 ± 0.55	−25.74 ± 0.48	−22.36 ± 0.09
*T*Δ*S* kJ mol^−1^	−2.42 ± 0.05	−0.35 ± 0.01	3.85 ± 0.08
*N*	0.78 ± 0.01	0.87 ± 0.01	1.45 ± 0.01

Host	β-CD^c^		

Guest	PAAADen	PAAADhn	PAAADddn

10^−3^*K* dm^3^ mol^−1^	8.77 ± 0.24	14.4 ± 0.06	5.77 ± 0.18
Δ*H* kJ mol^−1^	−20.81 ± 0.12	−15.45 ± 0.09	−16.58 ± 0.17
*T*Δ*S* kJ mol^−1^	1.72 ± 0.18	8.29 ± 0.18	4.89 ± 0.24
*N*	0.86 ± 0.07	0.85 ± 0.06	0.83 ± 0.05

^a^In aqueous Na_2_HPO_4_/KH_2_PO_4_ buffer at pH 7.0 and *I* = 0.10 mol dm^−3^. ^b^This study. ^c^Data from [3] obtained under identical conditions to those of this study. The errors shown are the fitting errors, and the experimental error is ≤5% in both studies.

The large *K* for the PAAβ-CDen/PAAADen and PAAβ-CDen/PAAADhn systems are mainly due to substantial Δ*H* contributions (Table 1). The negative *T*Δ*S* for the PAAβ-CDen/PAAADen system is attributable to the entropy loss arising from the combination of the β-CDen and ADen substituents into a single complex and network formation outweighing the entropy gain [49–50] anticipated for the displacement of water from the β-CDen annulus by the ADen substituent. This offset is smaller for the PAAβ-CDen/PAAADhn system possibly because the ADhn substituent hexyl tether allows more network flexibility. The *K* for the PAAβ-CDen/PAAADddn system is the largest observed as a consequence of a smaller Δ*H* being offset by a positive *T*Δ*S*. This probably arises from the entropy loss expected for complexation of the adamantyl group and the dodecyl tether of the ADddn substituent and network formation being outweighed by the entropy gain arising from displacement of water from the β-CDen annulus by the adamantyl group of ADddn and its dodecyl tether (under the titration conditions the solutions remain fluid whereas at higher concentrations the solution viscosity increases as the network formation becomes more extensive as discussed in the Rheological studies section).

A comparison with the complexation parameters for the β-CD/PAAADen, β-CD/PAAADhn and β-CD/PAAADddn systems from the literature (Table 1) shows these systems to be characterized by significantly smaller *K* and Δ*H* and more positive *T*Δ*S* [3]. (The *N =* 0.86 − 0.83 are also consistent with either the steric hindrance by the poly(acrylate) backbone, or the hydrophobic association of the adamantyl substituents, or both, hindering complexation). The greater stabilities of the PAAβ-CDen/PAAADen and PAAβ-CDen/PAAADhn systems are attributable to the cooperative stabilizing effect of network formation facilitated by β-CDen, ADen and ADhn being substituents on the poly(acrylate) backbone, and an accompanying decrease in entropy. The *N* = 1.45 and 0.83 (Table 1) for the PAAβ-CDen/PAAADddn and β-CD/PAAADddn systems, respectively, indicate the greater effect of the dodecyl tether on complexation in the first system by comparison with the second system where some complexation of the dodecyl tether occurs as indicated by ^1^H NMR spectroscopy but does not result in significant network formation [14].

### ^1^H NMR studies of substituted poly(acrylate) network formation

**Figure 5:**
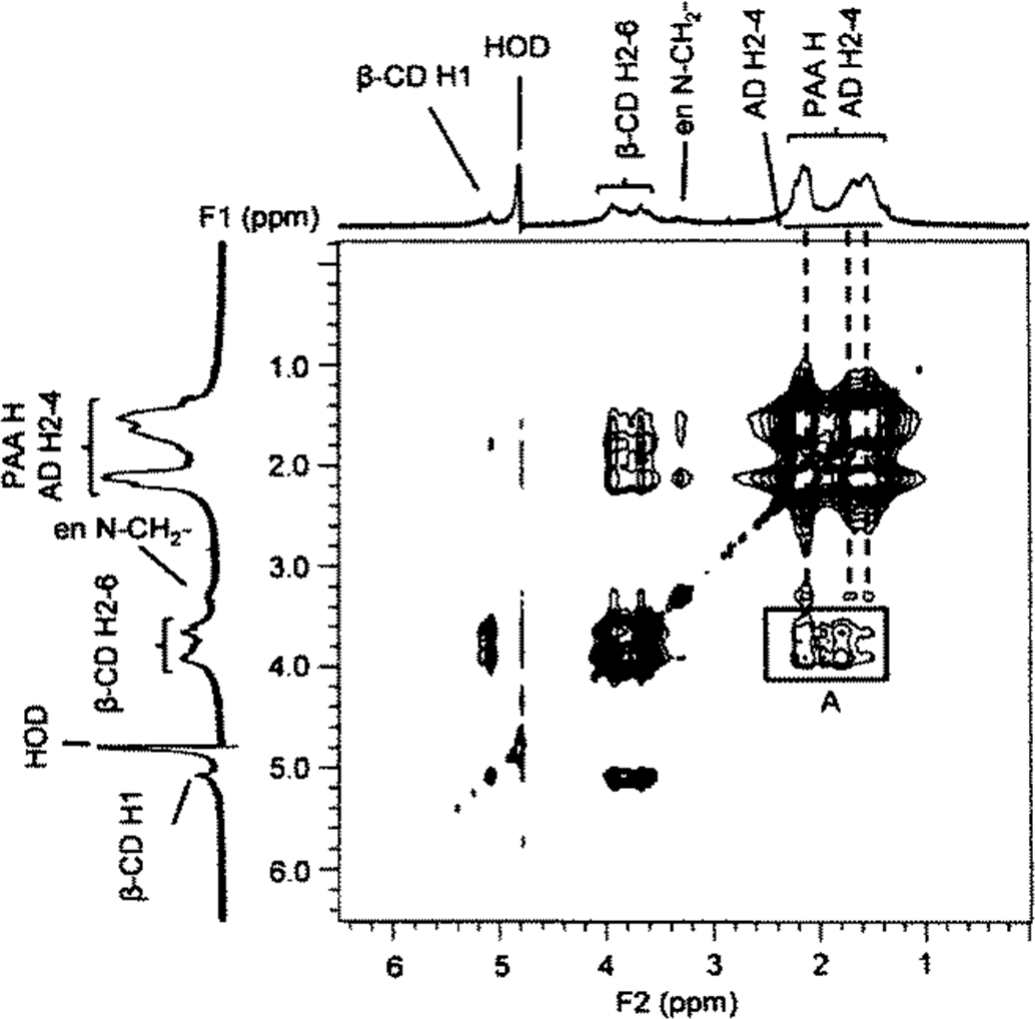
2D NOESY ^1^H NMR spectrum of 0.44 wt % PAAβ-CDen ([β-CDen] = 2.0 × 10^−3^ mol dm^−3^) and 0.60 wt % PAAADen ([ADen] = 2.0 × 10^−3^ mol dm^−3^) in D_2_O Na_2_HPO_4_/KH_2_PO_4_ buffer at pD 7.0 and *I* = 0.10 mol dm^−3^ at 298.2 K. The cross-peaks in box A are attributed to dipolar interactions between the annular H3,5,6 protons of the β-CD of the β-CDen substituents and the H2–4 protons of the ADen substituents.

Further insight into the complexation process is gained from the 2D NOESY ^1^H NMR spectrum of a D_2_O solution of PAAβ-CDen and PAAADen in which the β-CDen and ADen substituents are equimolar (Figure 5). Substitution of β-CD at C6^A^, as in PAAβ-CDen, renders all of the D-glucopyranose subunits inequivalent such that distinction between the H2–6 resonances is not possible as a consequence of small differences in the chemical shifts of the resonances of each D-glucopyranose subunit which results in a loss of definition in the β-CDen substituent spectrum. Thus, the cross-peaks in box A in Figure 5 cannot be unequivocally assigned to the anticipated dominant dipolar interactions between the β-CDen annular H3,5,6 protons and the H2–4 ADen protons of the host–guest complex, but they are consistent with such an interaction. The analogous spectrum for the PAAβ-CDen/PAAADhn system (Figure S2, Supporting Information File 1) also shows cross-peaks attributable to dipolar interactions between the β-CDen annular H3,5,6 protons and the H2–4 ADhn protons. (The 2D NOESY ^1^H NMR spectrum of PAAβ-CDen alone (Figure S3, Supporting Information File 1) shows no cross-peaks in the region where those assigned to β-CDen/ADen substituent dipolar interactions arise (Figure 5) indicating that β-CDen substituent proton dipolar interactions with PAAβ-CDen backbone protons are insignificant.) The similarity of the spectra in Figure 5 and Figure S2 (Supporting Information File 1) and the *K*, Δ*H*, *T*Δ*S* and *N* data (Table 1) for the PAAβ-CDen/PAAADen and PAAβ-CDen/PAAADhn systems is consistent with their host–guest interactions being similar in nature.

The 2D NOESY ^1^H NMR spectrum of a D_2_O solution of PAAβ-CDen and PAAADddn in which the β-CDen and ADddn substituents are equimolar shows cross-peaks arising from dipolar interactions between the annular H3,5,6 protons of the β-CDen substituent and also those of the H2–4 ADddn protons and the methylene protons of its dodecyl tether (Figure S4, Supporting Information File 1). This is consistent with complexation of both the adamantyl group and the dodecyl tether of the ADddn substituent by the β-CDen substituent, and thereby the likelihood of simultaneous complexation by two β-CDen substituents as previously discussed. (It has been reported that NOESY ^1^H NMR cross-peaks between the β-CD annular H3,H5,H6 annular protons and both the H2–4 ADddn protons and the methylene protons of its dodecyl tether are also observed for solutions of β-CD and PAAADddn [14].)

### UV–vis dye complexation studies

The complexations of the three dyes by β-CD alone in aqueous solution are used as a basis for assessing the effects of the substitution of β-CDen onto poly(acrylate) in PAAβ-CDen and the subsequent network formation with PAAADen, PAAADhn and PAAADddn on dye complexation. The variation of absorbance of all three dyes with added β-CD is consistent with the dominant formation of 1:1 β-CD–dye complexes (Equation 4 and Equation 5 where *A*, ε_dye_ and ε_β-CD–dye_ represent the observed absorbance and the molar absorbance of the dye and the β-CD–dye complex, respectively) as seen for EO, MO and MR in Figures S5–S7 (Supporting Information File 1), respectively. The wavelengths at which the maximum absorbances occur for the three dyes in their free and complexed states, λ_max_, and the derived *K*_11_ (Table 2) were determined by best-fitting an algorithm derived from Equations 4–6 to the titration absorbance data using a nonlinear least-squares program, HypSpec [51–52]. The largest and smallest *K*_11_ characterize the β-CD–EO and β-CD–MR host–guest complexes, respectively, and probably reflect the favorable stereochemistry of EO for complexation and its greater hydrophobicity.

[4]
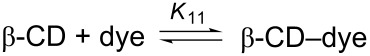


[5]
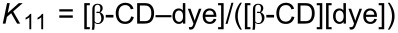


[6]
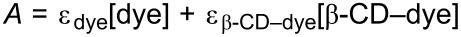


**Figure 6:**
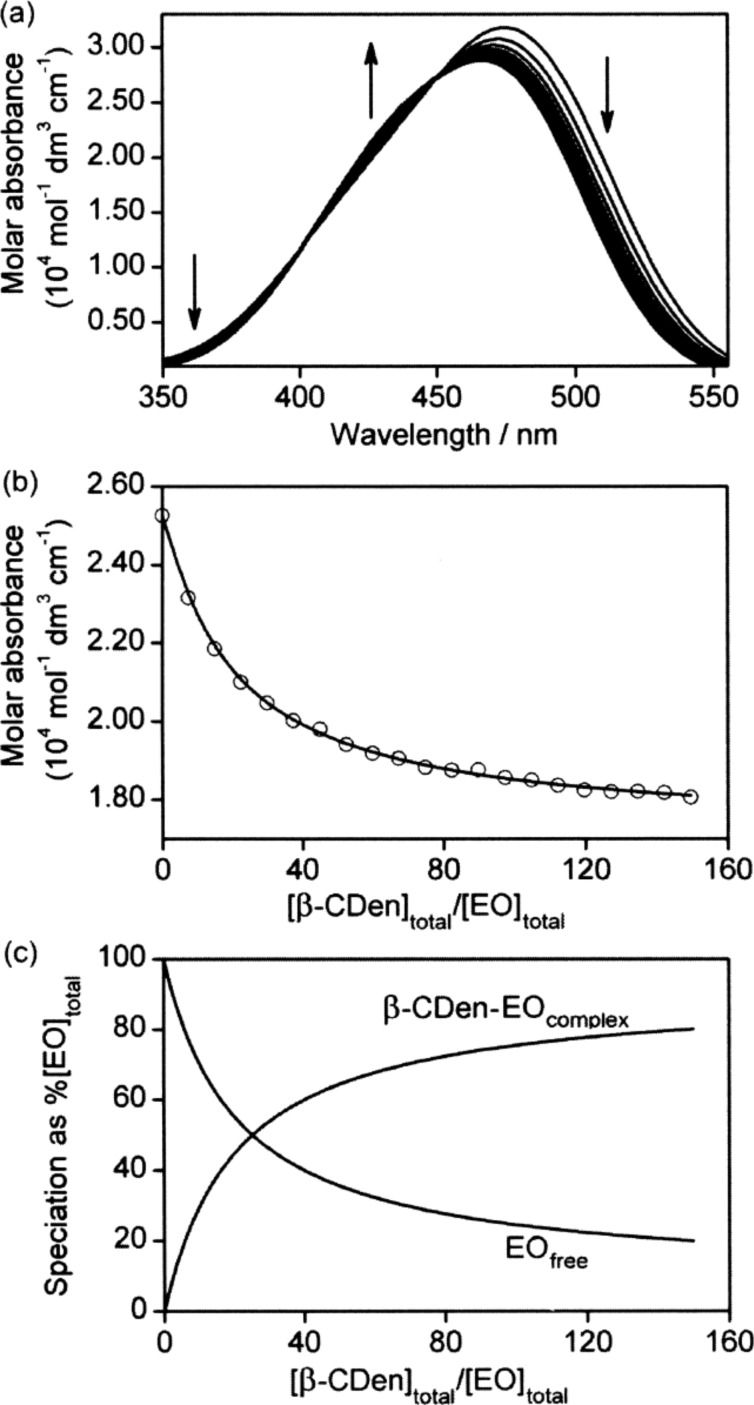
(a) Molar absorbance variation of 1.5 cm^3^ of a EO solution ([EO] = 2.00 × 10^−5^ mol dm^−3^) with 20 sequential additions of a PAAβ-CDen solution (50 mm^3^ each, 0.98 wt %, [β-CDen] = 4.49 × 10^−3^ mol dm^−3^) at 298.2 K. Both solutions were prepared in aqueous Na_2_HPO_4_/KH_2_PO_4_ buffer at pH 7.0 and *I* = 0.10 mol dm^−3^. The arrows indicate the direction of molar absorbance variation upon each addition of PAAβ-CDen solution. (b) Molar absorbance variation at 500 nm and the line representing the best fit of an algorithm for a 1:1 host–guest complexation of EO by β-CDen substituents of PAAβ-CDen over the wavelength range 475–525 nm. (c) Speciation plot with [EO]_total_ = 100% as the ratio [β-CDen]_total_/[EO]_total_ increases.

Systematic UV–vis absorbance changes also occur for the dyes upon addition of PAAβ-CDen as seen for EO in Figure 6 and MO and MR in Figures S8 and S9 (Supporting Information File 1), respectively. The *K*_11_, determined through an algorithm derived from equations analogous to Equations 4–6 in which β-CD is replaced by the β-CDen substituent, are substantially decreased in magnitude by comparison with those derived in the presence of β-CD, particularly for MR (Table 2). This is attributable to a combination of steric hindrance caused by the poly(acrylate) backbone and repulsion between the PAAβ-CDen carboxylate groups and the negatively charged dyes.

**Table 2:** Dye UV–vis absorption λ_max_ and complexation constants, *K*_11_, for complexation of MR, MO and EO by β-CD, β-CDen substituents of PAAβ-CDen and β-CDen substituents of PAAβ-CDen substituted poly(acrylate) in binary mixtures with PAAADen, PAAADhn or PAAADddn.

Host	Dye	Dye λ_max_ [nm]	*K*_11_^a^ [dm^3^ mol^−1^]

None	MR	430	–
	MO	464	–
	EO	474	–

β-CD	MR	414	772 ± 10
	MO	455	3255 ± 35
	EO	466	10515 ± 110

PAAβ-CDen	MR	415	76 ± 1
	MO	458	1454 ± 20
	EO	463	2230 ± 30

PAAβ-CDen/PAAADen	MR	–	–
	MO	457	1000 ± 10
	EO	463	1475 ± 20

PAAβ-CDen/PAAADhn	MR	–	–
	MO	457	875 ± 10
	EO	463	1411 ± 20

PAAβ-CDen/PAAADddn	MR	–	–
	MO	457	713 ± 10
	EO	467	986 ± 20

^a^In aqueous Na_2_HPO_4_/KH_2_PO_4_ buffer at pH 7.0 and *I* = 0.10 mol dm^−3^ at 298.2 K. The errors shown are fitting errors. The experimental error is ≤5%.

**Figure 7:**
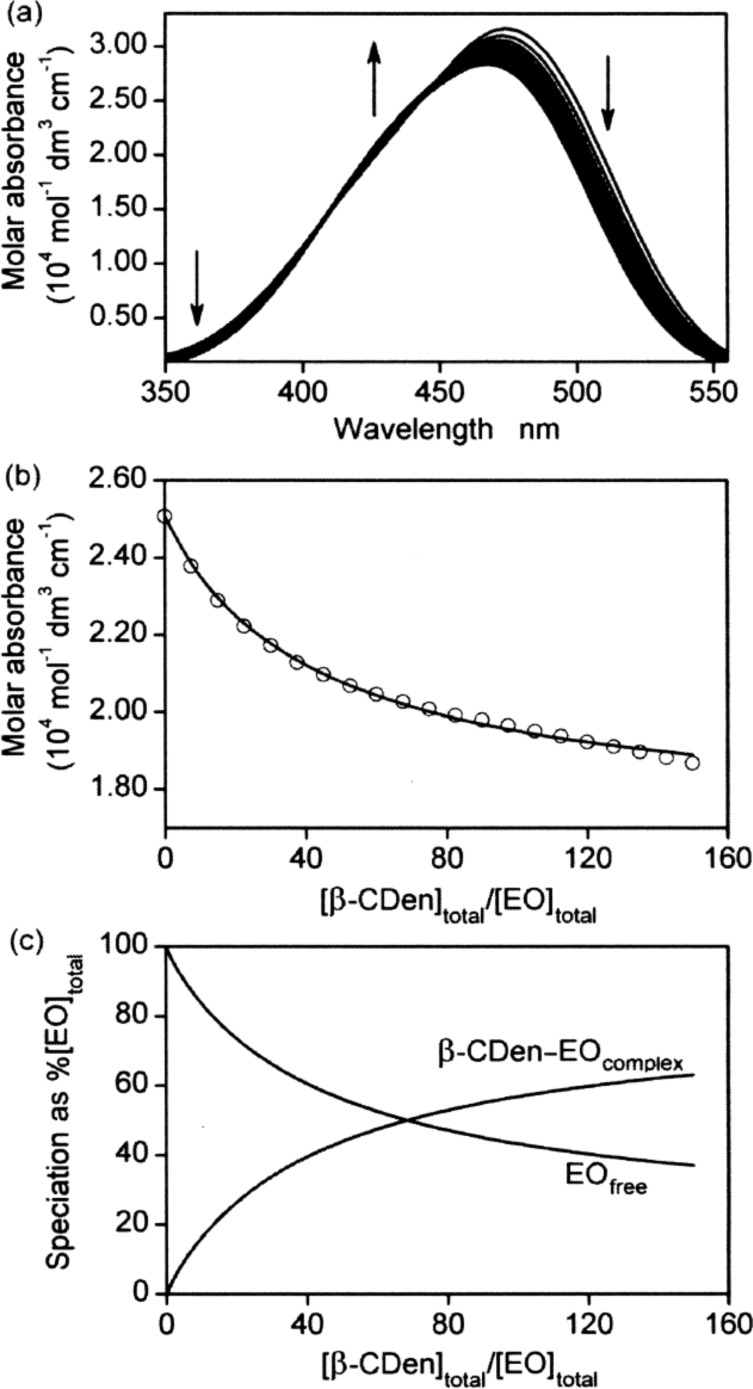
(a) Molar absorbance variation of 1.5 cm^3^ of a EO solution ([EO] = 2.00 × 10^−5^ mol dm^−3^) with 20 sequential additions of a PAAβ-CDen solution (25 mm^3^ each, 1.93 wt %, [β-CDen] = 9.03 × 10^−3^ mol dm^−3^) and a PAAADhn solution (25 mm^3^ each, 0.98 wt %, [ADhn] = 3.00 × 10^−3^ mol dm^−3^) at 298.2 K. All solutions were prepared in aqueous Na_2_HPO_4_/KH_2_PO_4_ buffer solutions at pH 7.0 and *I* = 0.10 mol dm^−3^. The arrows indicate the direction of molar absorbance variation upon each addition of the PAAβ-CDen and PAAADhn solutions. (b) Molar absorbance variation at 500 nm and the line representing the best-fit of an algorithm for a 1:1 host–guest complexation of EO by β-CDen substituents in the self-assembled PAAβ-CDen/PAAADhn network over the wavelength range 475–525 nm. (c) Speciation plot with [EO]_total_ = 100% as the ratio [β-CDen]_total_/[EO]_total_ increases.

The UV–vis variations observed for titration of the dyes with PAAβ-CDen and PAAADen, PAAADhn, or PAAADddn, as exemplified by the ternary PAAβ-CDen/PAAADhn/EO system (Figure 7), reflect the competition between the dye and the adamantyl substituent groups (and also the dodecyl tether for PAAADddn) and the dye for complexation by the β-CDen substituents of PAAβ-CDen, as do the derived *K*_11_ (Table 2). Thus, there are two competing equilibria for complexation by the β-CDen substituent in the PAAβ-CDen/PAAADhn network as shown in Equation 1 and Equation 7. The *K*_11_ for the host–guest complexation between the β-CDen substituents in the self-assembled PAAβ-CDen/PAAADhn network and the dye is defined by Equation 8 where [β-CDen], [dye] and [β-CDen–dye] represent the concentration of the β-CDen substituents in the PAAβ-CDen/PAAADhn network, the dye, and the dye complex at equilibrium, respectively. Given that [β-CDen]_total_ and [dye]_total_ are the total concentrations; mass balances are given by Equation 9 and Equation 10. The UV–vis absorbance at a particular wavelength is given by Equation 11 where *A*, ε_dye_ and ε_PAAβ-CDen–dye_ represent the observed absorbance and molar absorbance of the dye and the host–guest complex, respectively. The *K*_11_ (Table 2) were derived by best-fitting an algorithm based on Equations 1–3 and 7–11 to the UV–vis absorbance data using the HypSpec protocol [51–52].

[7]
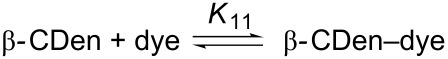


[8]
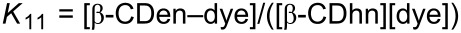


[9]
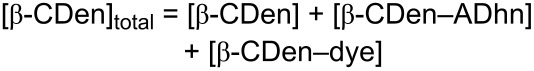


[10]
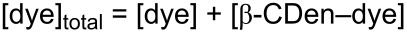


[11]
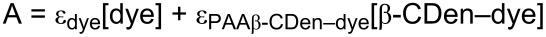


**Figure 8:**
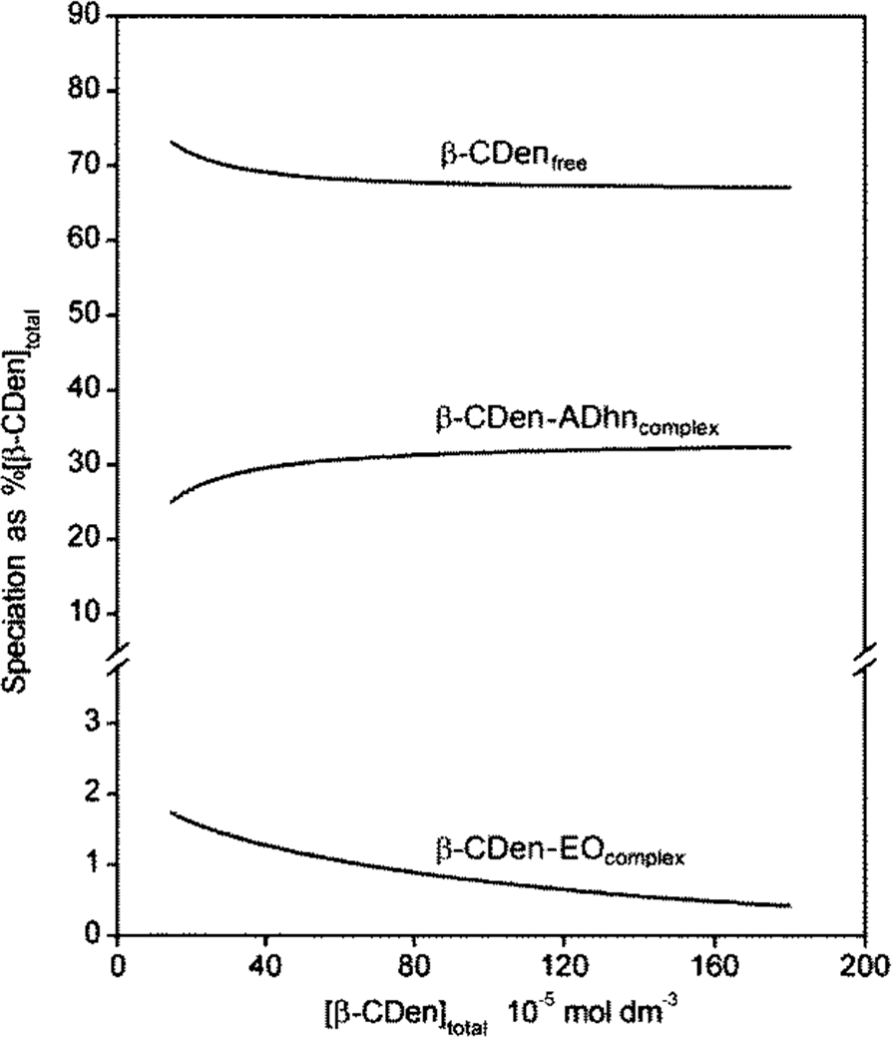
Speciation plot with [β-CDen]_total_ = 100% for the PAAβ-CDen/PAAADhn/EO system.

The variation of the β-CDen–EO complex concentration in the PAAβ-CDen/PAAADhn/EO network occurring during the course of titration is shown in Figure 7, and the accompanying changes in free β-CDen, β-CDen–ADhn complex and β-CDen–EO complex concentrations are shown in Figure 8. The analogous data for PAAβ-CDen/PAAADen/EO are quite similar (Figures S10 and S11, Supporting Information File 1) whereas those for PAAβ-CDen/PAAADddn/EO (Figures S12 and S13, Supporting Information File 1) differ considerably. The corresponding data for the PAAβ-CDen/PAAADen/MO, PAAβ-CDen/PAAADhn/MO and PAAβ-CDen/PAAADddn/MO systems appear in Figures S14–S19 (Supporting Information File 1). Collectively, these data facilitate determination of *K*_11_ for these six systems (Table 2) from which it is seen that in each case *K*_11_ is further decreased by comparison with that determined for complexation by β-CD and PAAβ-CDen, probably because of increased steric crowding within the network. The largest decreases in *K*_11_ occur for the PAAβ-CDen/PAAADddn/MO and PAAβ-CDen/PAAADddn/EO systems; decreases which may reflect the additional competition between the ADddn substituent dodecyl tether and the dyes for complexation by the β-CDen substituents. (The UV–vis absorbance changes observed for MR in the three networks (Figures S20–S22, Supporting Information File 1) are too small for derivation of *K*_11_ consistent with further decreases in *K*_11_ as also observed for the complexation of EO and MO in the analogous systems.)

**Figure 9:**
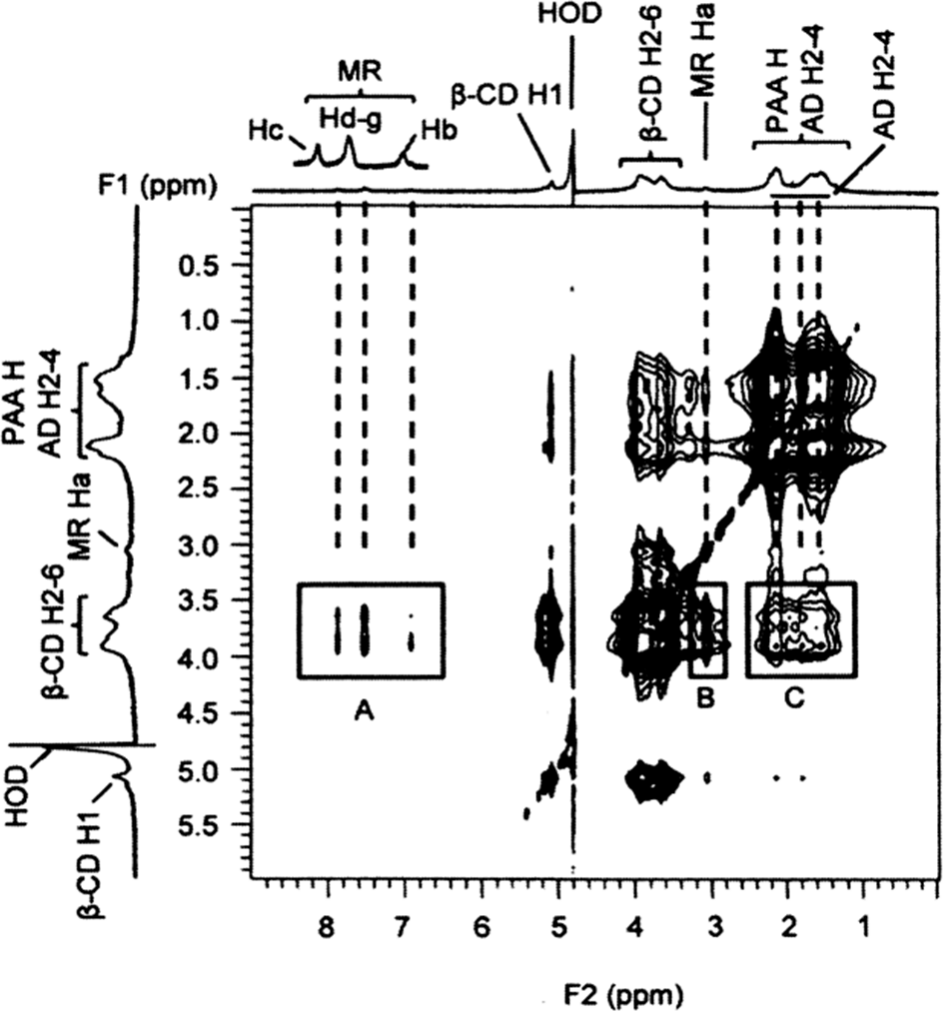
2D NOESY ^1^H NMR spectrum of MR ([MR] = 2.0 × 10^−3^ mol dm^−3^) in solution with PAAβ-CDen (0.78 wt %, [β-CDen] = 3.6 × 10^−3^ mol dm^−3^) and PAAADen (0.40 wt %, [ADen] = 1.2 × 10^−3^ mol dm^−3^) in D_2_O Na_2_HPO_4_/KH_2_PO_4_ buffer solution at pD 7.0 and *I* = 0.10 mol dm^−3^ at 298.2 K. Cross-peaks in boxes A and B are attributed to dipolar interactions of the annular H3,5,6 protons of β-CDen and the aromatic (Hb–g) and methyl (Ha) protons of MR, respectively. Cross-peaks in box C arise from dipolar interactions between the annular H3,5,6 protons of β-CDen and the H2–4 protons of ADen.

Equimolar D_2_O solutions of β-CD and each of the three dyes show 2D ^1^H ROESY NMR cross-peaks arising from dipolar interactions between the β-CD annular H3,5,6 protons and those of the dye (Figures S23–S25, Supporting Information File 1) consistent with dye complexation. The 2D ^1^H NOESY NMR spectra of solutions of PAAβ-CDen/dye, PAAβ-CDen/PAAADen/dye, PAAβ-CDen/PAAADhn/dye or PAAβ-CDen/PAAADddn/dye, where the β-CDen concentration is 3.6 × 10^−3^ mol dm^−3^, the adamantyl substituent concentration is 1.2 × 10^−3^ mol dm^−3^ in the last three systems and the dye concentration is 2.0 × 10^−3^ mol dm^−3^ show cross-peaks arising from dipolar interactions between the β-CDen annular H3,5,6 protons and those of the dye, as seen for the PAAβ-CDen/PAAADen/MR system in Figure 9 (analogous cross-peaks occur in the spectra for the other eleven systems as shown in Figures S26–S36, Supporting Information File 1), consistent with dye complexation in all twelve systems. Thus, despite the *K*_11_ for complexation of MR by the PAAβ-CDen/PAAADen, PAAβ-CDen/PAAADhn and PAAβ-CDen/PAAADddn networks being too small for reliable determination in the UV–vis studies, the observation of significant cross-peaks arising from dipolar interactions between the β-CDen substituent annular H3,5,6 protons and the MR Ha–g protons indicate the occurrence of complexation.

### Rheological studies

At higher solution concentrations than those studied by UV–vis spectroscopy, the networks formed by the three combinations: PAAβ-CDen/PAAADen, PAAβ-CDen/PAAADhn and PAAβ-CDen/PAAADddn separately and when complexing MR, MO or EO form hydrogels, the viscosities of which were determined by rheology. In each hydrogel [β-CDen] = 3.60 × 10^−3^ mol dm^−3^ and [ADen, ADhn or ADddn] = 1.20 × 10^−3^ mol dm^−3^, the concentration of each dye was 2.00 × 10^−3^ mol dm^−3^ and the overall concentration of substituted poly(acrylate)s was 1.14–1.20 wt %. (These hydrogel compositions are identical to those used in the dye release studies discussed below, and are presented in Table S1, Supporting Information File 1.) The viscosity variation of each system with the shear rate is shown for the binary systems PAAβ-CD/PAAADen, PAAβ-CD/PAAADhn and PAAβ-CD/PAAADddn in Figure S37 (Supporting Information File 1), and for the ternary systems in which each of the binary systems complexes the three dyes in Figure S38, Supporting Information File 1). The viscosities show small variations in the shear rate and those determined at 0.03 s^−1^ shear rate are selected for comparison purposes. (Because it was necessary to quantitatively determine the rates of dye release from these hydrogels, their fluidities must be sufficient to allow their quantitative transfer into the dye release measurement apparatus (Figure S39, Supporting Information File 1), and this determined their maximum component concentrations.)

**Figure 10:**
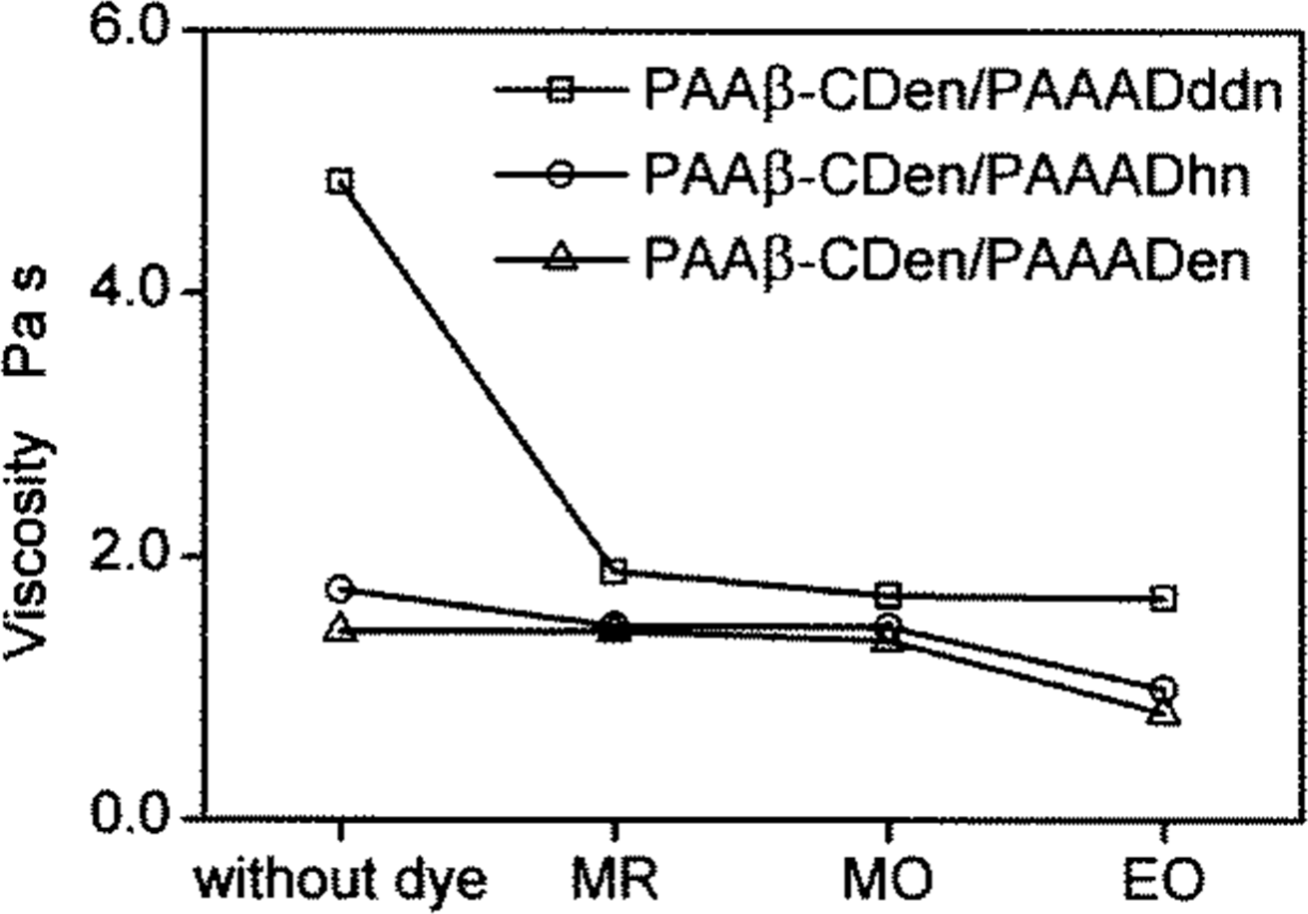
Viscosity variations at a 0.03 s^−1^ shear rate of 1.14 wt % PAAβ-CDen/PAAADen, 1.18 wt % PAAβ-CDen/PAAADhn, or 1.20 wt % PAAβ-CDen/PAAADddn in the absence and presence of MR, MO, or EO at 298.2 K in aqueous Na_2_HPO_4_/KH_2_PO_4_ buffer at pH 7.0 and *I* = 0.10 mol dm^−3^. In each system, the concentrations of the β-CDen and adamantyl substituents were 3.60 × 10^−3^ mol dm^−3^ and 1.20 × 10^−3^ mol dm^−3^, respectively, and the dye concentration was 2.00 × 10^−3^ mol dm^−3^.

The viscosity variations at a 0.03 s^−1^ shear rate for all twelve systems are shown in Figure 10 from which it is seen that, in the absence of dyes, the viscosities of the binary systems increase in the sequence: 1.14 w % PAAβ-CDen/PAAADen (1.44 Pa s) < 1.18 wt % 1.20 wt % PAAβ-CDen/PAAADhn (1.75 Pa s) < PAAβ-CDen/PAAADddn (4.85 Pa s). Thus, it appears that the two-fold β-CDen complexation of both the adamantyl group and the dodecyl tether of the ADddn substituent in the PAAβ-CDen/PAAADddn system (Figure 4), deduced to be present from the ITC and 2D NOESY ^1^H NMR studies, increases the viscosity and extent of network formation of the 1.20 wt % solutions of this system by comparison with those of the PAAβ-CDen/PAAADen and PAAβ-CDen/PAAADhn systems.

Upon addition of MR, MO or EO, a substantial decrease in the viscosities of the ternary PAAβ-CDen/PAAADddn/dye solutions occurs (viscosities = 4.85, 1.89, 1.71 and 1.59 Pa s in the presence of no dye, MR, MO and EO, respectively) whereas the changes in the 0.03 s^−1^ shear rate viscosities of the PAAβ-CDen/PAAADen (1.44, 1.49, 1.37 and 0.87 Pa s in the presence of no dye, MR, MO and EO, respectively) and PAAβ-CDen/PAAADhn (1.75, 1.48, 1.53 and 1.00 Pa s in the presence of no dye, MR, MO and EO, respectively) solutions are smaller. This is consistent with the dye complexation competing with that of the dodecyl tether of the ADddn substituent for a second β-CDen substituent in the PAAβ-CDen/PAAADddn/dye systems, even with the β-CDen substituent concentration being 1.8 times greater than that of the dyes, whereas this option is not available in the PAAβ-CDen/PAAADen/dye and PAAβ-CDen/PAAADhn/dye systems. Nevertheless, the 0.03 s^−1^ shear rate viscosities of the PAAβ-CDen/PAAADddn/dye systems are still greater than those of the PAAβ-CDen/PAAADen/dye and PAAβ-CDen/PAAADhn/dye systems consistent with some residual complexation of the dodecyl tether of the ADddn substituent and a consequent viscosity enhancement.

**Figure 11:**
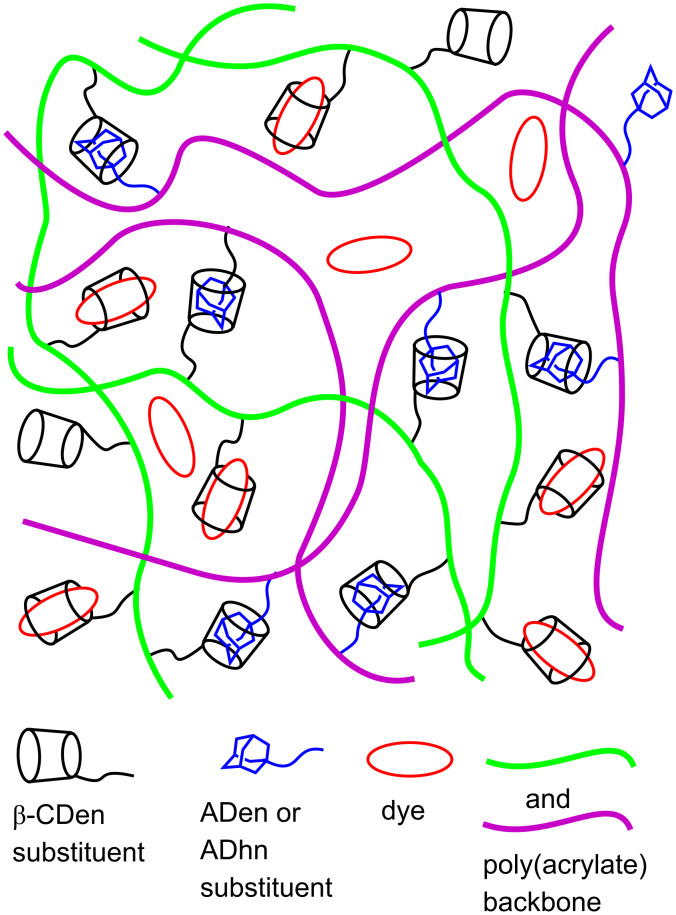
Representation of dye complexation in the PAAβ-CDen/PAAADen and PAAβ-CDen/PAAADhn networks.

The viscosities of the PAAβ-CDen/PAAADen/dye and PAAβ-CDen/PAAADhn/dye systems show little variation in the presence of MR and MO, but a small decrease occurs in the presence of EO consistent with it showing the largest *K*_11_ for both systems (as determined in the UV–vis studies) and competing more strongly with ADen and ADhn for complexation by β-CDen. The general picture which emerges for dye complexation in the PAAβ-CDen/PAAADen/dye and PAAβ-CDen/PAAADhn/dye systems is shown in Figure 11. In the PAAβ-CDen/PAAADddn/dye systems the dodecyl tether of the ADddn substituent is complexed by the β-CDen substituent as shown by 2D ^1^H NOESY NMR (Figures S34–S36, Supporting Information File 1) consistent with it competing with the dyes for complexation by β-CDen.

### Dye release studies

**Figure 12:**
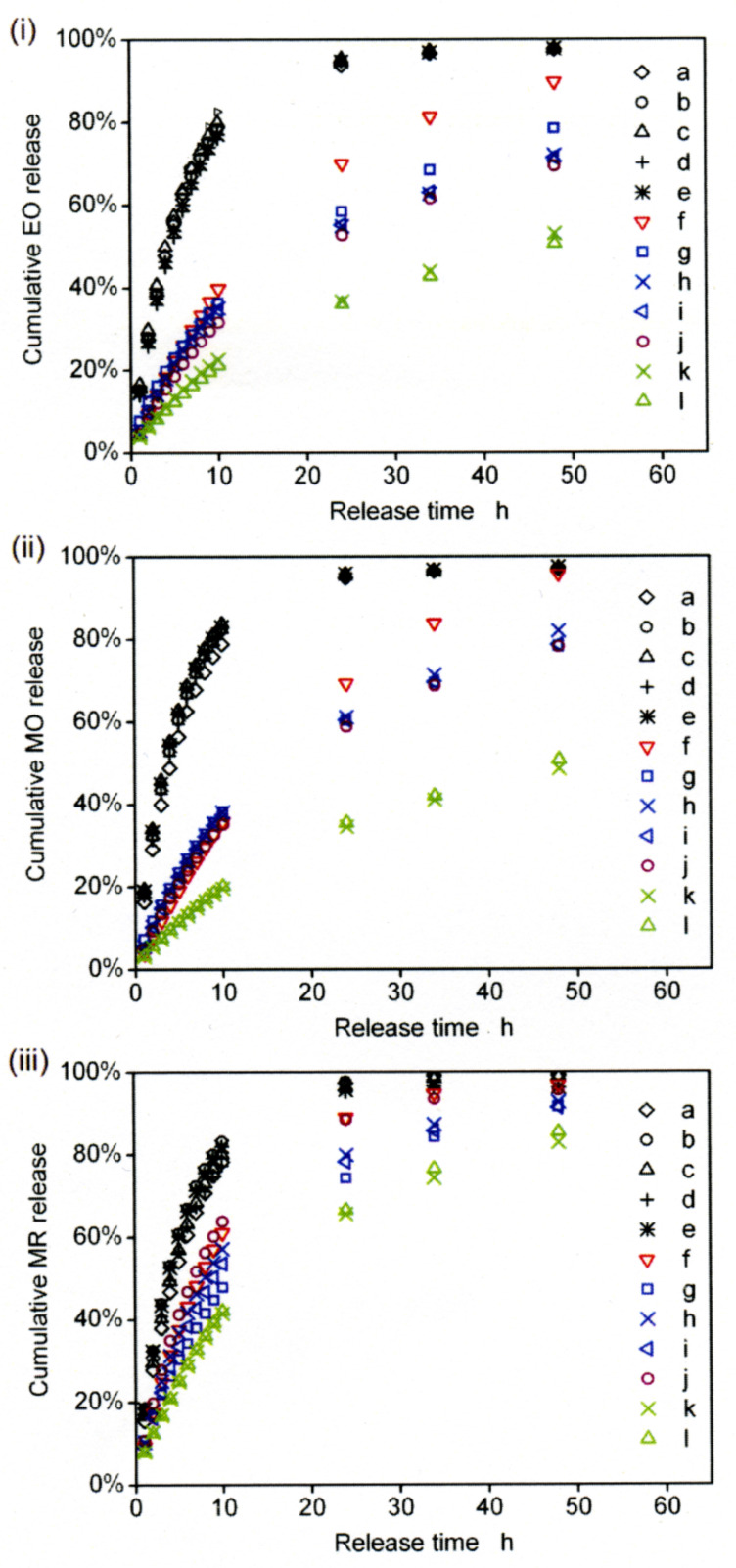
Release profiles for EO (i), MO (ii) and MR (iii) from aqueous (a) Na_2_HPO_4_/KH_2_PO_4_ buffer alone, (b) 1.20 wt % PAA, (c) 1.20 wt % PAA/PAAADddn, (d) 1.18 wt % PAA/PAAADhn, (e) 1.14 wt % PAA/PAAADen, (f) 1.20 wt % PAA/β-CD, (g) 1.20 wt % PAAβ-CDen/PAAADddn, (h) 1.18 wt % PAAβ-CDen/PAAADhn, (i) 1.14 wt % PAAβ-CDen/PAAADen, (j) 1.18 wt % PAA/PAAβ-CDen, (k) 1.96 wt % PAAβ-CDen/PAAADhn, and (l) 1.89 wt % PAAβ-CDen/PAAADen all in aqueous Na_2_HPO_4_/KH_2_PO_4_ buffer at pH 7.0, *I* = 0.10 mol dm^−3^ and 298.2 K. Initial [dye] = 2.00 × 10^−3^ mol dm^−3^.

Dye release through a dialysis membrane with pores allowing passage of species with a molecular weight up to 3.5 kDa into an aqueous Na_2_HPO_4_/KH_2_PO_4_ buffer at pH 7.0, *I* = 0.10 mol dm^−3^ and 298.2 K was characterized for each system. Reference solutions of MR, MO and EO were prepared in aqueous Na_2_HPO_4_/KH_2_PO_4_ buffer at pH 7.0 and *I* = 0.10 mol dm^−3^. All other dye solutions were prepared in the same buffer. To render the dye environment as similar as possible with respect to poly(acrylate), PAA, backbone concentration, those solutions containing neither PAAβ-CDen/PAAADen, PAAβ-CDen/PAAADhn nor PAAβ-CDen/PAAADddn had an appropriate amount of PAA added. The compositions of the thirty-six solutions studied appear in the caption to Figure 12 and in more detail in Table S1 (Supporting Information File 1.

The profiles of dye released with time for EO, MR and MO shown in Figure 12 exhibit informative trends. The solutions of EO in (a) Na_2_HPO_4_/KH_2_PO_4_ buffer alone, (b) 1.20 wt % PAA, (e) 1.14 wt % PAA/PAAADen, (d) 1.18 wt % PAA/PAAADhn and (c) 1.20 wt % PAA/PAAADddn show very similar release profiles (Figure 12(i)) with 90% of EO released within 20 h indicative of little interaction between EO and the other solutes. This is consistent with the major factors determining the appearance of EO in the receiving solution being its diffusion within the particular EO sample and its interaction with the dialysis membrane as it passes through its pores. Similar profiles characterize the release of MO and MR from PAA and adamantyl substituted PAA (Figure 12(ii) and 12(iii), respectively,) and a similar interpretation applies. However, the release of EO from a solution of (f) 1.20 wt % PAA/β-CD is slower reaching 87% after 48 h consistent with substantial formation of the β-CD–EO complex which, although of lower molecular weight than the membrane 3.5 kDa limit, is likely to pass through the membrane less readily than EO alone and is less mobile than free EO. An even slower release of EO from the (j) 1.18 wt % PAA/PAAβ-CDen solution occurs reaching only 69% after 48 h consistent with substantial formation of the β-CDen–EO complex within the PAAβ-CDen/EO solution which is of too high molecular weight to pass through the membrane such that the passage is limited to free EO alone. These data are consistent with complexation of EO by either β-CD alone or a β-CDen substituent within PAAβ-CDen controlling the amount of free EO in solution and thereby the rate of release through the membrane. A similar slowing of release is seen for the analogous MO and MR solutions (Figure 12(ii) and 12(iii)).

The EO release rate profiles for the (i) 1.14 wt % PAAβ-CDen/PAAADen, h) 1.18 wt % PAAβ-CDen/PAAADhn and (g) 1.20 wt % PAAβ-CDen/PAAADddn networks fall between those for the (f) 1.20 wt % PAA/β-CD and (j) 1.18 wt % PAA/PAAβ-CDen solutions consistent with EO complexation by the β-CDen substituents being of major importance in controlling the rate of EO release. The release of EO is significantly more rapid from the (g) 1.20 wt % PAAβ-CDen/PAAADddn solution than from the (h) 1.18 wt % PAAβ-CDen/PAAADhn and (i) 1.14 wt % PAAβ-CDen/PAAADen solutions, but this variation is much less for MR and MO (Figure 12(ii) and 12(iii)). This coincides with the (g) 1.20 wt % PAAβ-CDen/PAAADddn/EO solution being substantially more viscous than the (h) 1.18 wt % PAAβ-CDen/PAAADhn/EO and (i) 1.14 wt % PAAβ-CDen/PAAADen/EO solutions whereas the viscosities of the three corresponding MR and MO solutions are more similar (Figure 10). This indicates that viscosity is a significant rate determining factor for dye release.

The effect of increasing network extension and viscosity on the rate of EO release is illustrated by the profiles for the more viscous (l) 1.89 wt % PAAβ-CDen/PAAADen and (k) 1.96 wt % PAAβ-CDen/PAAADhn solutions from which 50% of EO is released in 48 h, and is attributable to an increase in β-CDen substituent concentration increasing the proportion of EO complexed and the more extensive network slowing free EO movement. A slowing of dye release for the analogous MO and MR solutions (Figure 12(ii) and 12(iii), respectively) is similarly explained. (A 1.90 wt % PAAβ-CDen/PAAADddn solution was too viscous to transfer quantitatively to the release apparatus.)

While the release of MR in (a) Na_2_HPO_4_/KH_2_PO_4_ buffer alone, (b) 1.20 wt % PAA, (e) 1.14 wt % PAA/PAAADen, (d) 1.18 wt % PAA/PAAADhn and (c) 1.20 wt % PAA/PAAADddn solutions (Figure 12(iii)) show profiles similar to those observed for EO (Figure 12(i)), the profiles for MR release from the (f) 1.20 wt % PAA/β-CD solution and the six solutions, (g)–(l), containing PAAβ-CDen are compressed into a shorter time-frame of more rapid release than that observed for the corresponding EO solutions. This is consistent with the weaker complexing of MR by comparison with EO (Table 2) and the higher proportion of free MR facilitating more rapid release. The release profiles for MO (Figure 12(ii)) more closely resemble those of EO as anticipated from the strength of complexing of MO being between that of EO and MR (Table 2).

The release rates of the three dyes from their (j) 1.18 wt % PAA/PAAβ-CDen/dye, (i) 1.14 wt % PAAβ-CDen/PAAADen/dye and (h) 1.18 wt % PAAβ-CDen/PAAADhn/dye solutions decrease in the order MR > MO > EO (Figures S40–S42, Supporting Information File 1) consistent with the same order of decrease in *K*_11_ for dye complexation (Table 2) largely determining the relative release rates. While the release of MR from the (g) 1.20 wt % PAAβ-CDen/PAAADddn/dye solution is the most rapid, the release profiles of MO and EO are more similar (Figure S43, Supporting Information File 1). This indicates that the dodecyl tether of the ADddn substituent diminishes the effect of the magnitude of *K*_11_ on the relative release rates of MO and EO possibly as a result of variations in the network structure as MO and EO compete with the dodecyl tether for complexation in the β-CDen substituent annuli. Finally, while the rate of MR release from the (f) 1.20 wt % PAA/β-CD/dye solution (Figure S44, Supporting Information File 1) is the most rapid, the two analogous but less rapid release profiles for MO and EO overlap which probably reflects a combination of the abilities of MO, EO and the β-CD–MO and β-CD–EO complexes to pass through the pores of the dialysis membrane.

## Conclusion

The self-assembly of the β-CDen and ADen, ADhn and ADddn substituted poly(acrylate) networks, PAAβ-CDen/PAAADen, PAAβ-CDen/PAAADhn and PAAβ-CDen/PAAADddn, and their complexation and release of the dyes, ethyl orange, methyl orange and methyl red have been characterized in aqueous solution. The factors controlling the dye release from these networks are the strength of complexation of the dye, which is dependent upon the structure of the dye, and the structure of the network and its viscosity. Potentially, these systems form the basis for the development of controlled drug delivery systems for topical and wound applications, where the factors for drug release are likely to be similar to those controlling the dye release.

## Experimental

### Materials

The sodium salts of methyl red (BDH), methyl orange (BDH), ethyl orange (Sigma-Aldrich), and β-cyclodextrin (Nihon Shokuhin Kako Co.) were used as received. Poly(acrylic acid) (*M*_w_ = 250 kDa, *M*_w_/*M*_n_ ≈ 2) was purchased from Aldrich as a 35 wt % aqueous solution and freeze-dried to a constant weight. The substituted poly(acrylate)s: PAAβ-CDen [15], PAAADen [13], PAAADhn [17] and PAAADddn [17] (Figure 1) were synthesized according to literature procedures, and the extent of random substitution of poly(acrylate) with the β-CDen, ADen, ADhn and ADddn substituents was determined from their ^1^H NMR spectra to be 8.8 ± 0.2%, 3.3 ± 0.1%, 3.0 ± 0.1% and 2.9 ± 0.1%, respectively.

### NMR spectroscopy

A Varian Inova 600 spectrometer operating at 599.96 MHz was used to run 1D, 2D NOESY and 2D ROESY ^1^H NMR spectra using standard pulse sequences with a mixing time of 0.3 s in the last two cases. All sample solutions were prepared in D_2_O Na_2_HPO_4_/KH_2_PO_4_ buffer solutions at pD 7.0 and *I* = 0.10 mol dm^−3^ and were equilibrated at the thermostated probe temperature of 298.2 ± 0.1 K for 30 min in 5 mm diameter NMR tubes prior to their spectra being recorded. Chemical shifts (δ, ppm) were internally referenced to HOD at δ = 4.79 ppm. The substitution percentage of the β-CDen substituents on the PAAβ-CDen backbone was determined from a comparison of the resonance areas of the β-CD H1 protons and the poly(acrylate) protons. The substitution percentages of the adamantyl substituents on the PAAADen, PAAADhn and PAAADddn backbones were determined through a comparison of the resonance area of the methylene protons of both -NHCH_2_ tether groups with that of the superimposed resonance areas of the poly(acrylate), adamantyl H2–4 and other tether methylene protons. The compositions of all solutions appear in the corresponding figure captions.

### Isothermal titration calorimetry (ITC)

ITC measurements were made using a MicroCal VP isothermal titration calorimeter. In each titration, 10 mm^3^ aliquots of a 0.62 wt % PAAβ-CDen solution ([β-CDen] = 2.84 × 10^−3^ mol dm^−3^) were titrated into 1.46 cm^3^ of either a 0.062 wt % PAAADen ([ADen] = 2.06 × 10^−4^ mol dm^−3^), 0.064 wt % PAAADhn ([ADhn] = 1.91 × 10^−4^ mol dm^−3^), or 0.072 wt % PAAADddn ([ADddn] = 2.03 × 10^−4^ mol dm^−3^) solution at 298.2 K using a computer-controlled micro-syringe at intervals of 210 s. All solutions were prepared in aqueous Na_2_HPO_4_/KH_2_PO_4_ buffer at pH 7.0 and *I* = 0.10 mol dm^−3^. The heats of dilution were determined by titrating aqueous Na_2_HPO_4_/KH_2_PO_4_ buffer (pH 7.0 and *I* = 0.10 mol dm^−3^) into similarly buffered PAAADen, PAAADhn and PAAADddn solutions and by titrating similarly buffered PAAβ-CDen solution into the buffer solution. The heats of dilution were subtracted from the total heats evolved to give the heats of host–guest complexation from which the complexation constant, *K*, and the corresponding Δ*H*, *T*Δ*S* and *N* were calculated using the Origin 7.0 MicroCal protocol [53] as described in the Results and Discussion section*.*

### UV–vis spectroscopy

The UV–vis spectra were recorded with a Cary-Varian 5000 UV–vis spectrophotometer using 1 cm path length matched quartz cells. All UV–vis titrations were performed in aqueous Na_2_HPO_4_/KH_2_PO_4_ buffer at pH 7.0 and *I* = 0.10 mol dm^−3^ at 298.2 K. For the β-CD/dye titrations, 50 mm^3^ aliquots of a β-CD solution were sequentially titrated into 1.5 cm^3^ of each dye solution and 1.5 cm^3^ of each reference solution. The UV–vis absorbance spectra were recorded prior to and after each of 20 sequential additions of β-CD solution. The PAAβ-CDen/dye titrations where carried out in a similar manner using the same solution volumes. For the PAAβ-CDen/adamantyl-substituted PAA/dye studies, a 25 mm^3^ aliquot of a PAAβ-CDen solution followed by 25 mm^3^ aliquots of a PAAADen, PAAADhn, or PAAADddn solution were sequentially titrated in a twenty-fold sequence into 1.5 cm^3^ of each dye solution and 1.5 cm^3^ of each reference solution. The UV–vis absorbance spectra were recorded prior to and after each of the twenty sequential additions of PAAβ-CDen solution and a PAAADen, PAAADhn, or PAAADddn solution. The concentrations of all solutions used in the UV–vis titrations are given in the figure captions. The complexation constants for dye complexation, *K*_11_, and the corresponding Δ*H* and *T*Δ*S* were derived from the UV–vis data as described in the Results and Discussion section.

### Rheology

Rheological measurements were carried out with a Physica MCR 501 (Anton Parr GmbH) stress-controlled rheometer with a 25 mm cone and plate geometry. The temperature was controlled at 298.2 K by a Peltier plate. All solutions were prepared in aqueous Na_2_HPO_4_/KH_2_PO_4_ buffer at pH 7.0 and *I* = 0.10 mol dm^−3^. The compositions of sample solutions are shown in the corresponding figure captions.

### Dye release

Dye release studies were performed using a membrane diffusion apparatus (Figure S37, Supporting Information File 1) in which a 3.5 kDa molecular weight cut-off dialysis membrane (Spectr/Por 3) of surface area 7.0 cm^2^ separated 5.0 cm^3^ of the dye containing solution (made up in Na_2_HPO_4_/KH_2_PO_4_ buffer at pH 7.0 and *I* = 0.10 mol dm^−3^ with MR, MO or EO concentration = 2.00 × 10^−3^ mol dm^−3^) from 200 cm^3^ of aqueous Na_2_HPO_4_/KH_2_PO_4_ buffer at pH 7.0 and *I* = 0.10 mol dm^−3^ which acted as the receiving solution. During the release experiments, the receiving solution was stirred at 298.2 K. At appropriate time intervals, a 2.0 cm^3^ sample of the receptor solution was withdrawn, its UV–vis spectrum was measured and the sample was then returned to the receiving solution. The dye concentration in the receiving solution was calculated by reference to its molar absorbance spectrum determined under the same conditions. Each dye solution composition is given in Table S1 (Supporting Information File 1). All solutions were stirred and equilibrated at 298.2 K before dye release measurements commenced.

## Supporting Information

File 1 Additional titrations, spectra and data.
