# Supplementary material for: β-Cyclodextrin- and adamantyl-substituted poly(acrylate) self-assembling aqueous networks designed for controlled complexation and release of small molecules
Source: Beilstein J Org Chem. 2017 Sep 7;13:1879–92. doi: 10.3762/bjoc.13.183 (PMC5629389; doi:10.3762/bjoc.13.183)
Supplement: File 1 — Additional titrations, spectra and data. [file Beilstein_J_Org_Chem-13-1879-s001.pdf]

Supporting Information

for

β-Cyclodextrin- and adamantyl-substituted poly(acrylate) self-assembling aqueous networks designed for controlled complexation and release of small molecules

Liang Yan[1], Duc-Truc Pham[1], Philip Clements[1], Stephen F. Lincoln*[1], Jie Wang[2], Xuhong Guo*[2], and Christopher J. Easton[3]

Address: [1]Department of Chemistry, University of Adelaide, Adelaide, SA 5005, Australia, [2]State Key Laboratory of Chemical Engineering, East China University of Science and Technology, Shanghai 200237, China and [3]Research School of Chemistry, Australian National University, Canberra, ACT 0200, Australia

Email: Stephen F. Lincoln* - stephen.lincoln@adelaide.edu.au; Xuhong Guo* - guoxuhong@ecust.edu.cn

* Corresponding author

Additional titrations, spectra and data

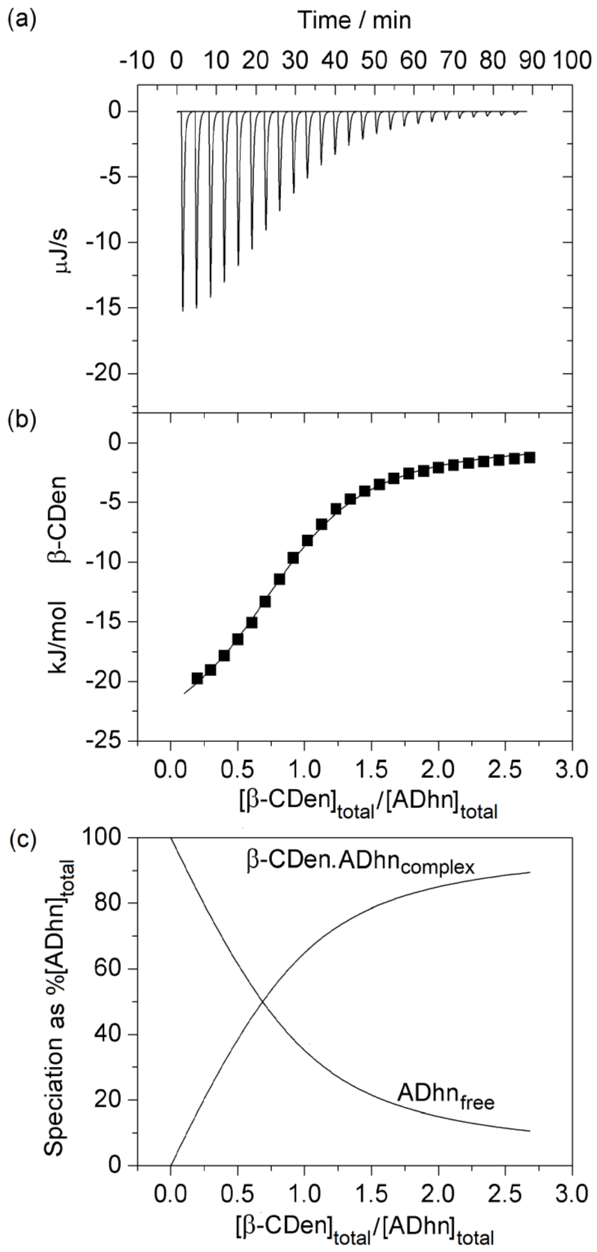

Figure S1: ITC data for the PAAβ-CDen/PAAADhn system obtained in aqueous Na_2HPO_4/KH_2PO_4 pH 7.0 buffer at I = 0.10 mol dm^{-3}. (a) Titration of 10 mm^3 aliquots of a 0.62 wt % PAA*β*-CDen ([β-CDen] = 2.84 × 10^{-3} mol dm^{-3}) into 1.46 cm^3 of 0.072 wt % PAAADen ([ADhn] = 2.03 × 10^{-4} mol dm^{-3}). (b) The solid curve shows the best fit of an algorithm for host-guest complexation between the β-CDen and ADhn substituents to the titration data. (c) Speciation plot showing the variation of [β-CDen–ADhn]$_{complex}$ and of [ADhn]$_{free}$ as a percentage of [ADhn]$_{total}$ = 100%.

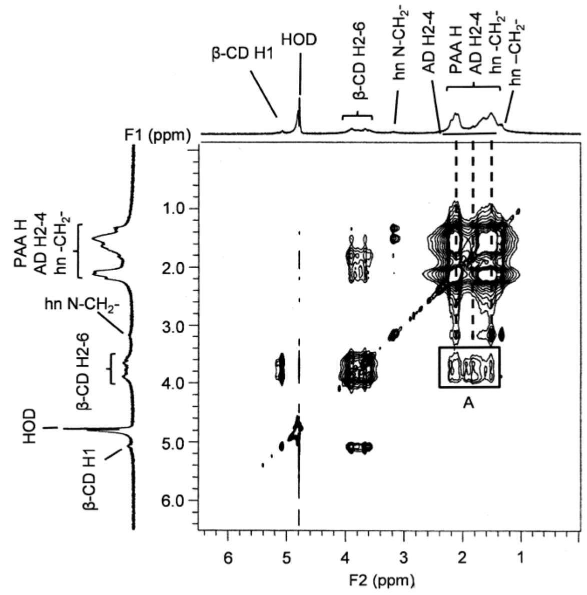

Figure S2: 2D NOESY ^{1}H NMR spectrum of 0.44 wt % PAAβ-CDen ([β-CDen] = 2.0 × 10^{-3} mol dm^{-3}) and 0.67 wt % PAAADhn ([ADhn] = 2.0 × 10^{-3} mol dm^{-3}) in D_2O Na_2HPO_4/KH_2PO_4 buffer at pD = 7.0 and I = 0.10 mol dm^{-3} at 298.2 K. The cross-peaks in box A are attributed to dipolar interactions the annular H3,5,6 protons of the β-CD of the β-CDen substituents and the H2–4 protons of the ADhn substituents.

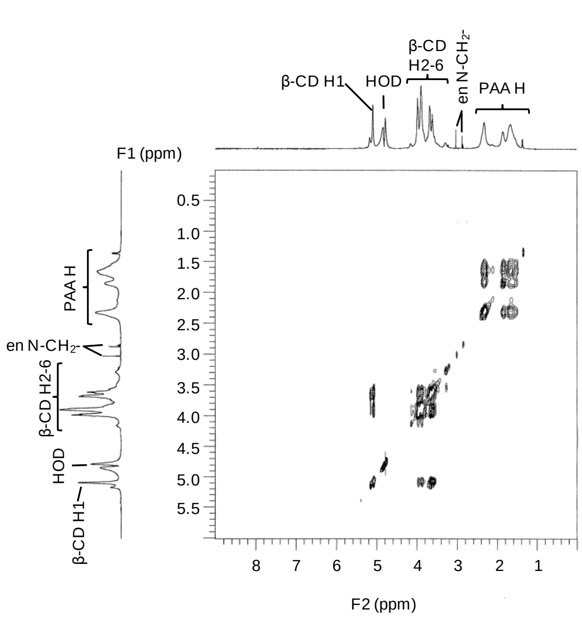

Figure S3: 2D NOESY ^{1}H NMR spectrum of 0.44 wt % PAAβ-CDen ([β-CDen] = 2.0 × 10^{-3} mol dm^{-3}) in D_2O Na_2HPO_4/KH_2PO_4 buffer solution at pD = 7.0 and I = 0.10 mol dm^{-3} at 298.2 K.

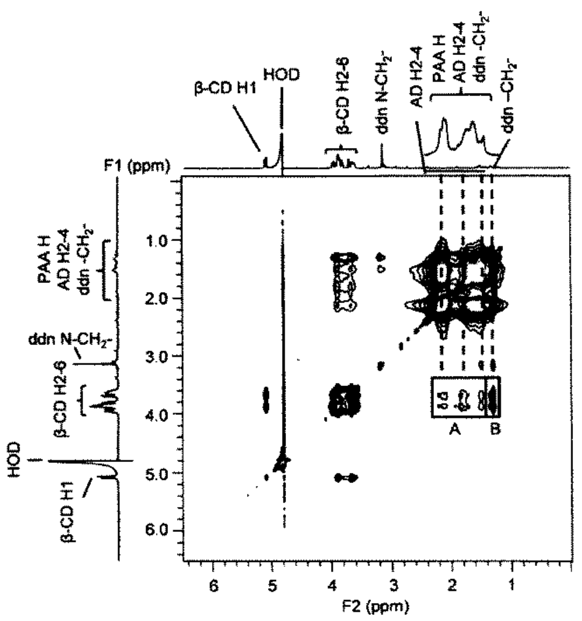

Figure S4: 2D NOESY ^{1}H NMR spectrum of 0.44 wt % PAAβ-CDen ([β-CDen] = 2.0 × 10^{-3} mol dm^{-3}) and 0.70 wt % PAAADddn ([ADddn] = 2.0 × 10^{-3} mol dm^{-3}) in D_2O Na_2HPO_4/KH_2PO_4 buffer at pD = 7.0 and I = 0.10 mol dm^{-3} at 298.2 K. The cross-peaks in box A are attributed to dipolar interactions between the annular H3,5,6 protons of the β-CD of the β-CDen substituents and the H2-4 protons of the ADddn substituents. Cross-peaks in box B are attributed to dipolar interactions between the annular H3,5,6 protons of the β-CDen substituents and the tether dodecyl methylene protons (ddn-CH_2-).

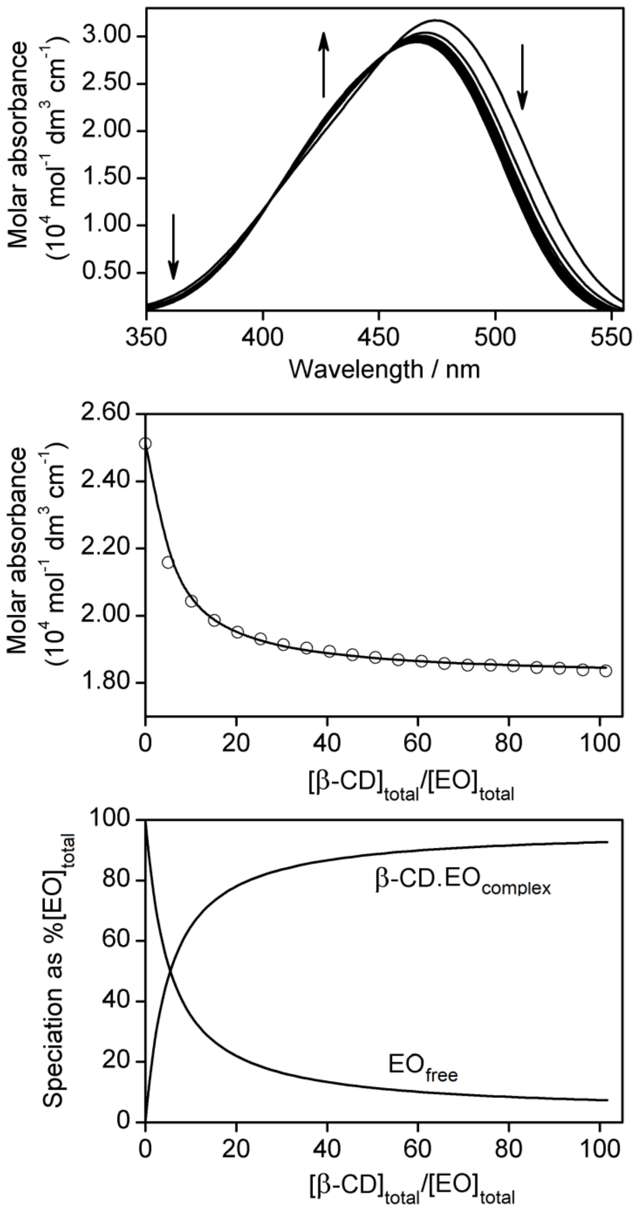

Figure S5: (Top) Molar absorbance variation of 1.5 cm^3 of an EO solution ([EO] = 2.00 × 10^{-5} mol dm^{-3}) with 20 sequential additions of a β-CD solution (50 mm^3 each, [β-CD] = 3.04 × 10^{-3} mol dm^{-3}) at 298.2 K. Both solutions were prepared in aqueous Na_2HPO_4/KH_2PO_4 buffer at pH 7.0 and I = 0.10 mol dm^{-3}. The arrows indicate the direction of molar absorbance change with each addition of the β-CD solution. (Middle) Molar absorbance variation at 500 nm and the line representing the best-fit of an algorithm for a 1:1 host-guest complexation of EO by β-CD over the wavelength range 475–525 nm. (Bottom) Speciation plot with [EO]$_{total}$ = 100% as the ratio [β-CD]$_{total}$/[EO]$_{total}$ increases.

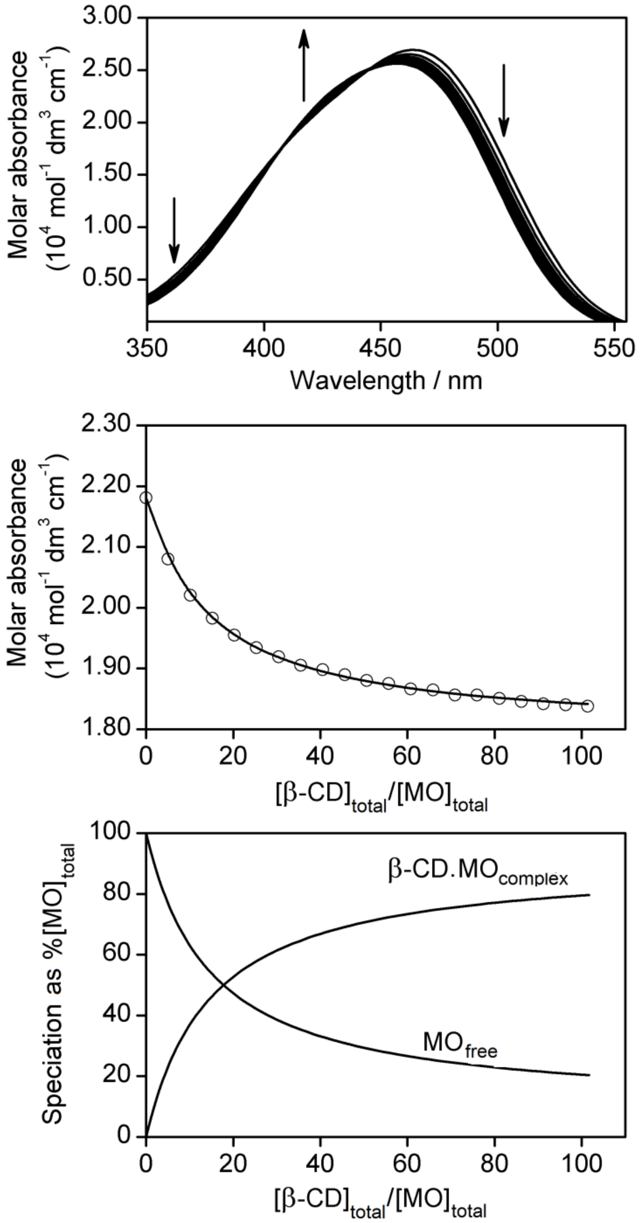

Figure S6: (Top) Molar absorbance variation of 1.5 cm^3 of a MO solution ([MO] = 2.00 × 10^{-5} mol dm^{-3}) with 20 sequential additions of a β-CD solution (50 mm^3 each, [β-CD] = 3.04 × 10^{-3} mol dm^{-3}) at 298.2 K. Both solutions were prepared in aqueous Na_2HPO_4/KH_2PO_4 buffer at pH 7.0 and I = 0.10 mol dm^{-3}. The arrows indicate the direction of molar absorbance variation with each addition of the β-CD solution. (Middle) Molar absorbance variation at 490 nm and the line representing the best-fit of an algorithm for a 1:1 host–guest complexation of MO by β-CD over the wavelength range 465–515 nm. (Bottom) Speciation plot with $[MO]_{total}$ = 100% as the ratio $[\beta\text{-CD}]_{total}/[MO]_{total}$ increases.

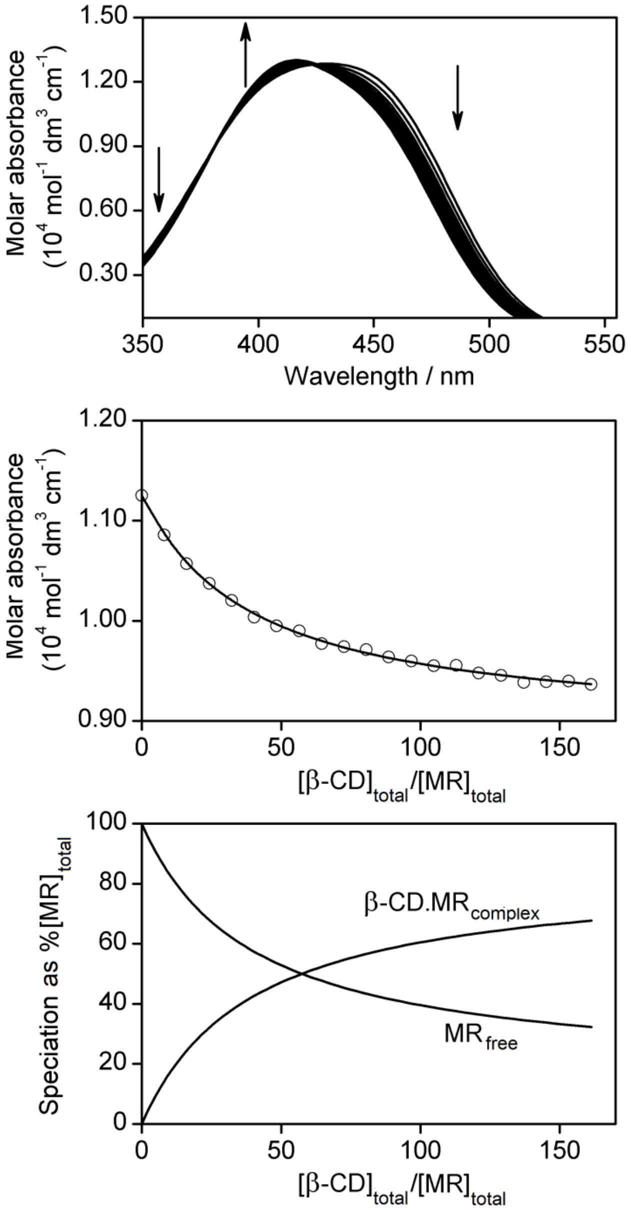

Figure S7: (Top) Molar absorbance variation of 1.5 cm^3 of a solution of MR ([MR] = 2.80 × 10^{-5} mol dm^{-3}) with 20 sequential additions of a β-CD solution (50 mm^3 each, [β-CD] = 6.77 × 10^{-3} mol dm^{-3}) at 298.2 K. Both solutions were prepared in aqueous Na_2HPO_4/KH_2PO_4 buffer at pH 7.0 and I = 0.10 mol dm^{-3}. The arrows indicate the direction of molar absorbance change with each addition of the β-CD solution. (Middle) Molar absorbance variation at 460 nm and the line representing the best-fit of an algorithm for a 1:1 host–guest complexation of MR by β-CD over the wavelength range 430–480 nm. (Bottom) Speciation plot with $[MR]_{total}$ = 100% as the ratio $[β\text{-CD}]_{total}/[MR]_{total}$ increases.

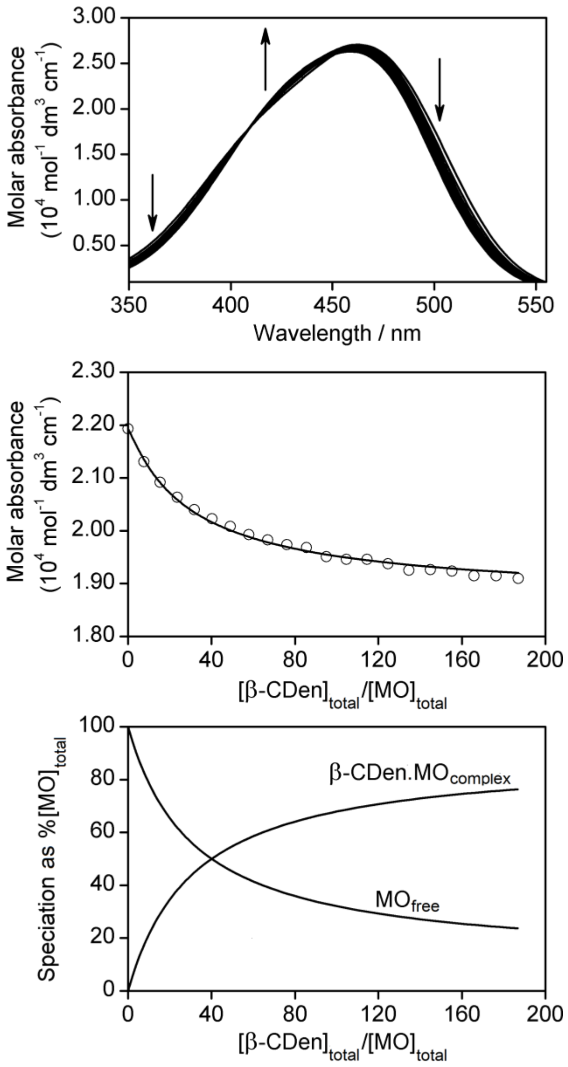

Figure S8: (Top) Molar absorbance variation of a MO solution ([MO] = 2.00×10^{-5} mol dm^{-3}) upon 20 sequential additions of a PAAβ-CDen solution (50 mm^3 each, 0.98 wt %, [β-CDen substituents] = 4.50×10^{-3} mol dm^{-3}) at 298.2 K. Both solutions were prepared in aqueous Na_2HPO_4/KH_2PO_4 buffer at pH 7.0 and $I = 0.10$ mol dm^{-3}. The arrows indicate the direction of molar absorbance variation upon each addition of the PAAβ-CDen solution. (Middle) Molar absorbance variation at 490 nm and the line representing the best-fit of an algorithm for a 1:1 host–guest complexation of MO by β-CDen substituents of PAAβ-CDen over the wavelength range 465–515 nm. (Bottom) Speciation plot with $[MO]_{total}$ = 100% as the ratio $[β\text{-CDen}]_{total}/[MO]_{total}$ increases.

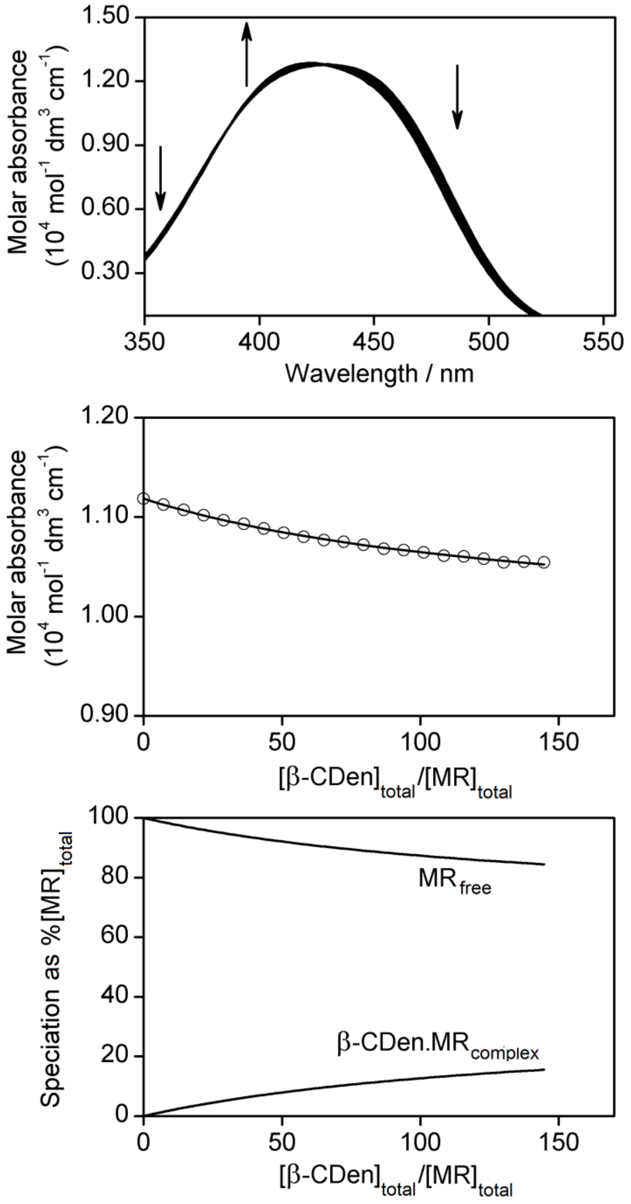

Figure S9: (Top) Molar absorbance variation of a MR solution ([MR] = 2.80×10^{-5} mol dm^{-3}) with 20 sequential additions of a PAAβ-CDen solution (50 mm^3 each, 1.32 wt %, [β-CDen] = 6.10×10^{-3} mol dm^{-3}) at 298.2 K. Both solutions were prepared in aqueous Na_2HPO_4/KH_2PO_4 buffer at pH 7.0 and I = 0.10 mol dm^{-3}. The arrows indicate the direction of molar absorbance variation with each addition of the PAAβ-CDen solution. (Middle) Molar absorbance variation at 460 nm and the line representing the best-fit of an algorithm for a 1:1 host–guest complexation of MR by β-CDen substituents of PAAβ-CDen over the wavelength range 430–480 nm. (Bottom) Speciation plot with $[MR]_{total}$ = 100% as the ratio $[β\text{-CDen}]_{total}/[MR]_{total}$ increases.

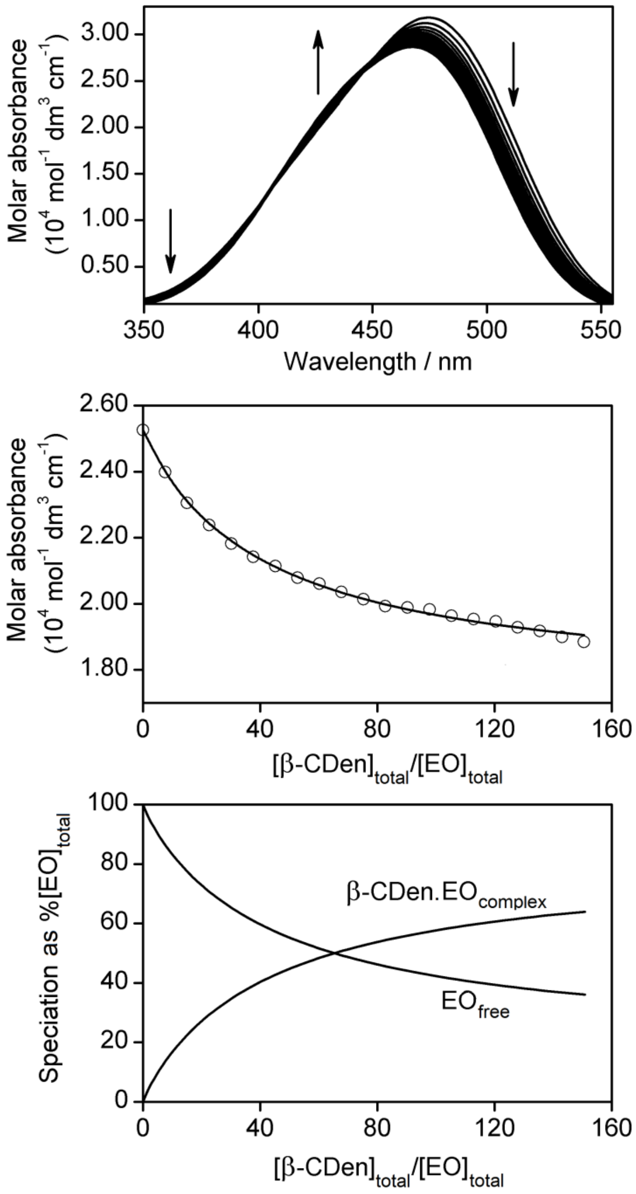

Figure S10: (Top) Molar absorbance variation of a 1.5 cm^3 of a solution of EO ([EO] = 2.00 × 10^{-5} mol dm^{-3}) with 20 sequential additions of a PAAβ-CDen solution (25 mm^3 each, 1.93 wt %, [β-CDen] = 9.03 × 10^{-3} mol dm^{-3}) and a PAAADen solution (25 mm^3 each, 0.91 wt %, [ADen] = 3.09 × 10^{-3} mol dm^{-3}) at 298.2 K. All solutions were prepared in aqueous Na_2HPO_4/KH_2PO_4 buffer solutions at pH 7.0 and I = 0.10 mol dm^{-3}. The arrows indicate the direction of molar absorbance variation with each addition of the PAAβ-CDen and PAAADen solutions. (Middle) Molar absorbance variation at 500 nm and the line representing the best-fit of an algorithm for a 1:1 host–guest complexation of EO by β-CDen substituents in self-assembled PAAβ-CDen/PAAADen network over the wavelength range 475–525 nm. (Bottom) Speciation plot with $[EO]_{total}$ = 100% as the $[β\text{-CDen}]_{total}/[EO]_{total}$ increases.

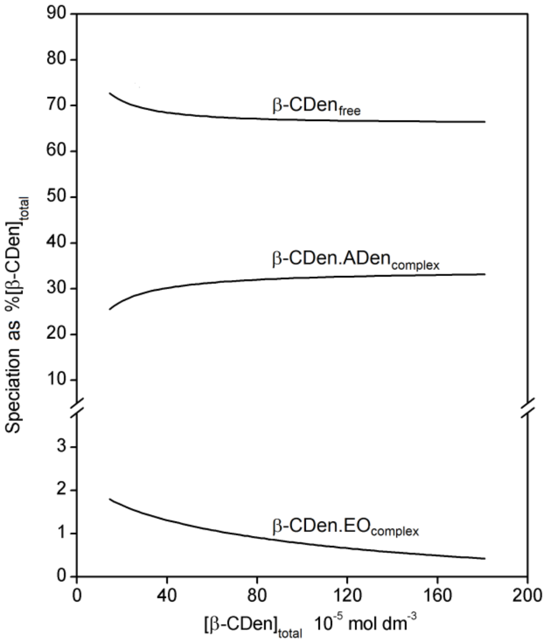

Figure S11: Speciation plot with [β-CDen]$_{total}$ = 100% for the PAAβ-CDen/PAAADen/EO system.

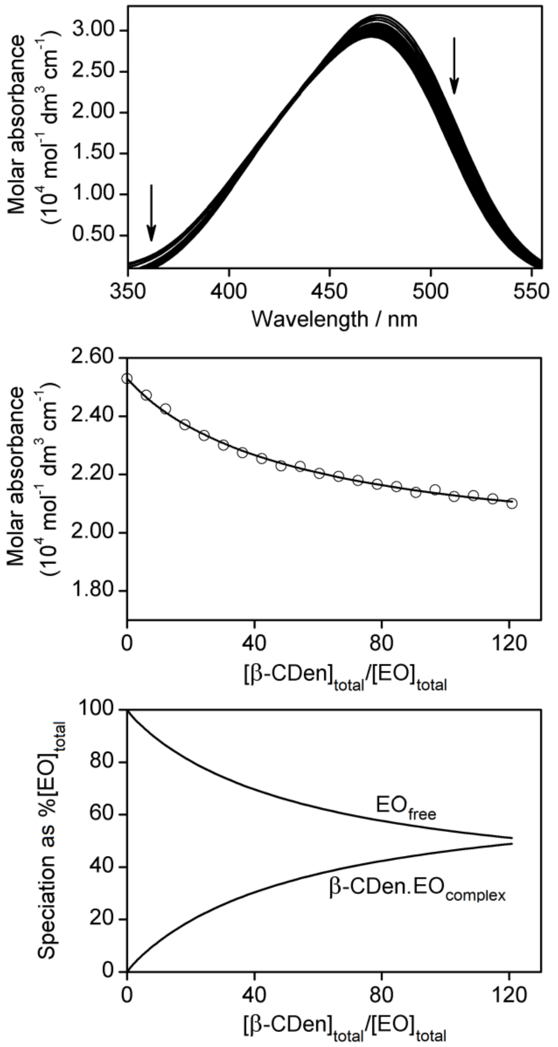

Figure S12: (Top) Molar absorbance variation of 1.5 cm^3 of a solution of EO ([EO] = 2.00 × 10^{-5} mol dm^{-3}) with 20 sequential additions of a PAAβ-CDen solution (25 mm^3 each, 1.56 wt %, [β-CDen] = 7.26 × 10^{-3} mol dm^{-3}) and a PAAADddn solution (25 mm^3 each, 0.85 wt %, [ADddn] = 2.43 × 10^{-3} mol dm^{-3}) at 298.2 K. All solutions were prepared in aqueous Na_2HPO_4/KH_2PO_4 buffer at pH 7.0 and I = 0.10 mol dm^{-3}. The arrows indicate the direction of molar absorbance variation with each addition of the PAAβ-CDen and PAAADddn solutions. (Middle) Molar absorbance variation at 500 nm and the line representing the best-fit of an algorithm for a 1:1 host–guest complexation of EO by β-CDen substituents in self-assembled PAAβ-CDen/PAAADddn network over the wavelength range 475–525 nm. (Bottom) Speciation plot with [EO]$_{total}$ = 100% as the[β-CDen]$_{total}$/[EO]$_{total}$ increases.

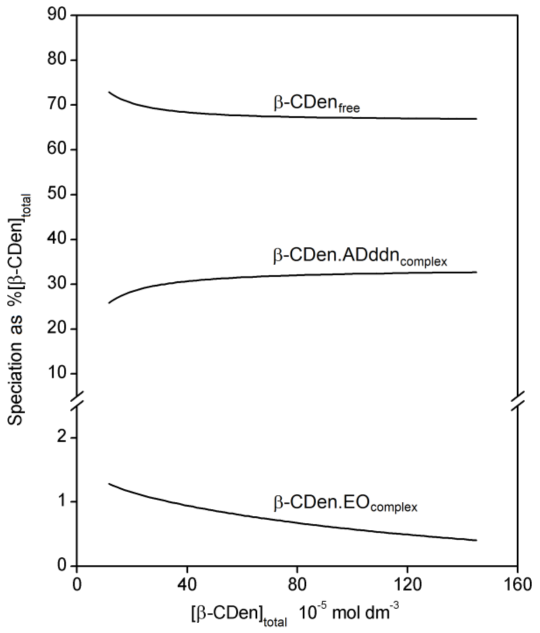

Figure S13: Speciation plot with [β-CDen]$_{total}$ = 100% for PAAβ-CDen/PAAADddn/EO system.

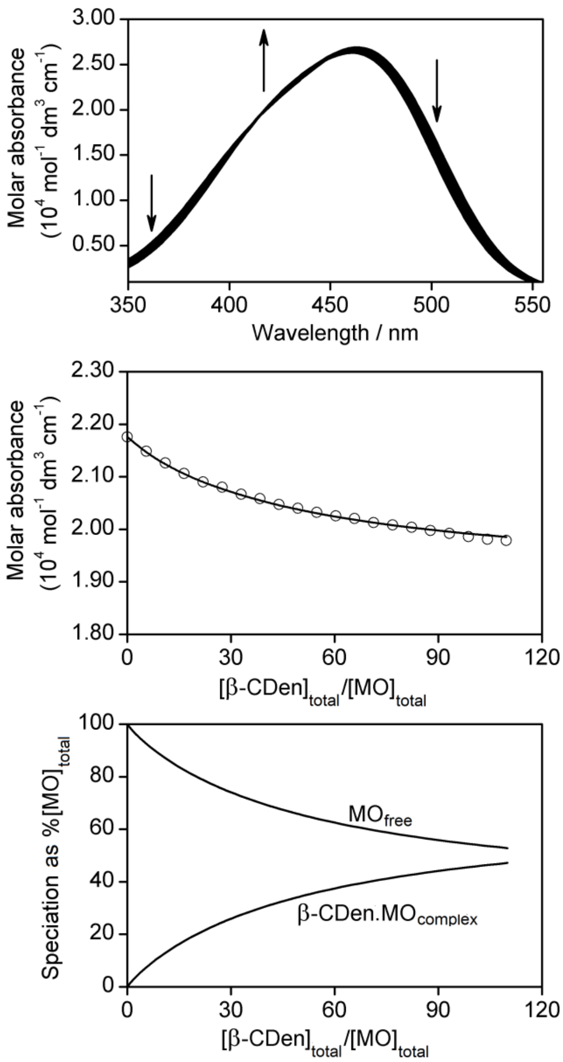

Figure S14: (Top) Molar absorbance variation of 1.5 cm^3 of a solution of MO ([MO] = 2.00 × 10^{-5} mol dm^{-3}) upon 20 sequential additions of a PAAβ-CDen solution (25 mm^3 each, 1.42 wt %, [β-CDen] = 6.58 × 10^{-3} mol dm^{-3}) and a PAAADen solution (25 mm^3 each, 0.65 wt %, [ADen substituents] = 2.19 × 10^{-3} mol dm^{-3}) at 298.2 K. All solutions were prepared in aqueous Na_2HPO_4/KH_2PO_4 buffer solutions at pH 7.0 and I = 0.10 mol dm^{-3}. The arrows indicate the direction of molar absorbance variation upon each addition of the PAAβ-CDen and PAAADen solutions. (Middle) Molar absorbance variation at 490 nm and the line representing the best-fit of an algorithm for a 1:1 host–guest complexation of MO by β-CDen substituents in self-assembled PAAβ-CDen/PAAADen network over the wavelength range 465–515 nm. (Bottom) Speciation plot with $[MO]_{total}$ = 100% as the $[\beta\text{-CDen}]_{total}/[EO]_{total}$ increases.

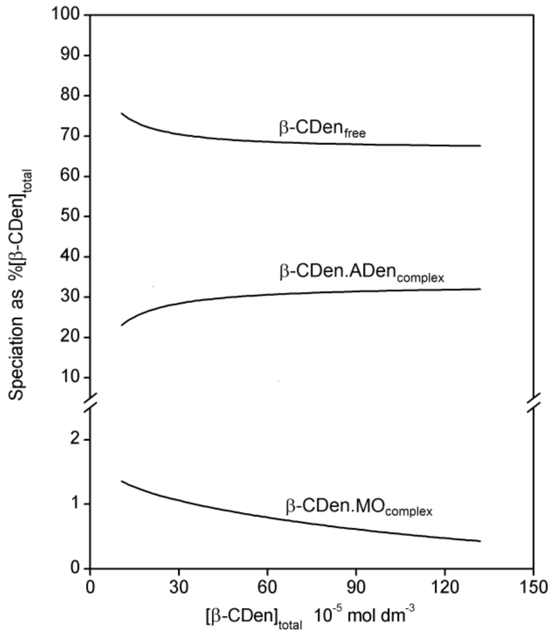

Figure S15: Speciation plot with [β-CDen]$_{total}$ = 100% for the PAAβ-CDen/PAAADen/MO system.

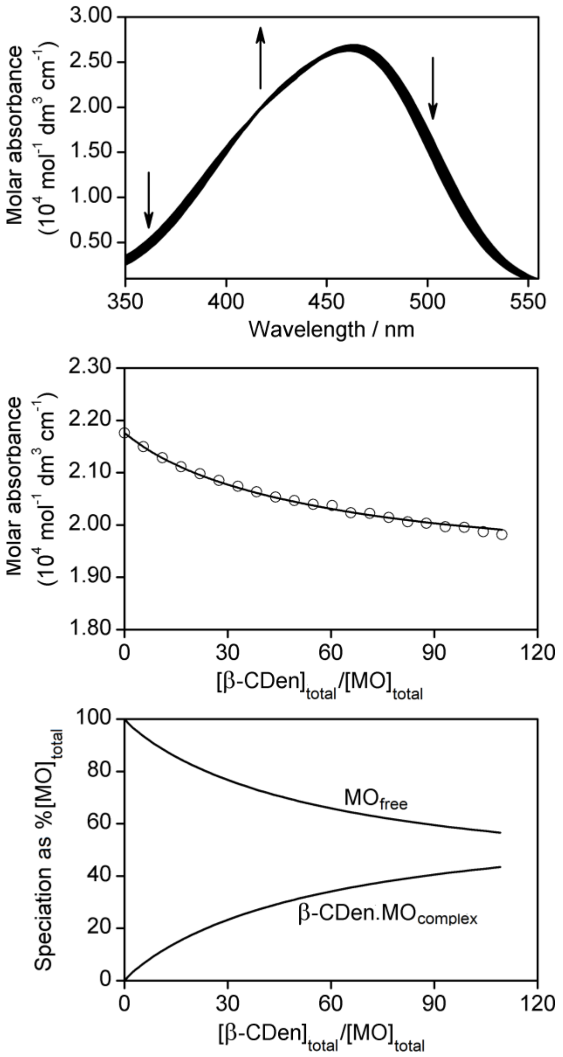

Figure S16: (Top) Molar absorbance variation of 1.5 cm^3 of a solution of MO ([MO] = 2.00 × 10^{-5} mol dm^{-3}) with 20 sequential additions of a PAAβ-CDen solution (25 mm^3 each, 1.42 wt %, [β-CDen] = 6.58 × 10^{-3} mol dm^{-3}) and a PAAADhn solution (25 mm^3 each, 0.74 wt %, [ADhn] = 2.23 × 10^{-3} mol dm^{-3}) at 298.2 K. All solutions were prepared in aqueous Na_2HPO_4/KH_2PO_4 buffer at pH 7.0 and I = 0.10 mol dm^{-3}. The arrows indicate the direction of molar absorbance variation with each addition of the PAAβ-CDen and PAAADhn solutions. (Middle) Molar absorbance variation at 490 nm and the line representing the best-fit of an algorithm for a 1:1 host–guest complexation of MO by β-CDen substituents in self-assembled PAAβ-CDen/PAAADhn network over the wavelength range 465–515 nm. (Bottom) Speciation plot with $[MO]_{total}$ = 100% as the $[β\text{-CDen}]_{total}/[MO]_{total}$ increases.

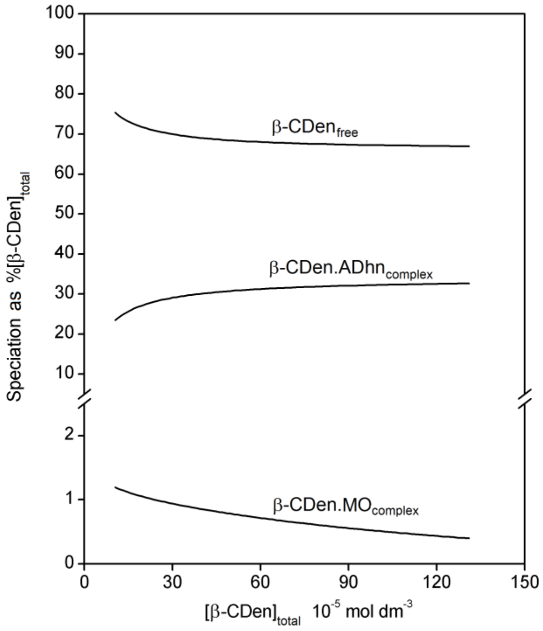

Figure S17: Speciation plot with $[\beta\text{-CDen}]_{total}$ = 100% for the PAAβ-CDen/PAAADhn/MO system.

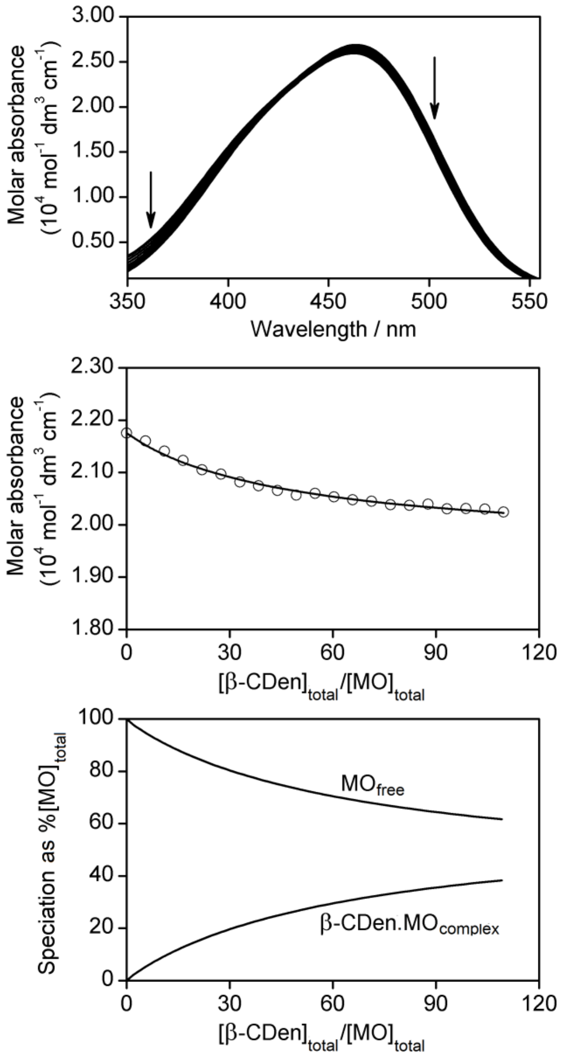

Figure S18: (Top) Molar absorbance variation of 1.5 cm^3 of a solution of MO ([MO] = 2.00×10^{-5} mol dm^{-3}) with 20 sequential additions of a PAAβ-CDen solution (25 mm^3 each, 1.42 wt %, [β-CDen] = 6.58×10^{-3} mol dm^{-3}) and a PAAADddn solution (25 mm^3 each, 0.78 wt %, [ADddn] = 2.23×10^{-3} mol dm^{-3}) at 298.2 K. All solutions were prepared in aqueous Na_2HPO_4/KH_2PO_4 buffer at pH 7.0 and I = 0.10 mol dm^{-3}. The arrows indicate the direction of molar absorbance variation each addition of the PAAβ-CDen and PAAADddn solutions. (Middle) Molar absorbance variation at 490 nm and the line representing the best-fit of an algorithm for a 1:1 host–guest complexation of MO by β-CDen substituents in self-assembled PAAβ-CDen/PAAADddn network over the wavelength range 465–515 nm. (Bottom) Speciation plot with $[MO]_{total}$ = 100% as the $[β\text{-CDen}]_{total}/[MO]_{total}$ increases.

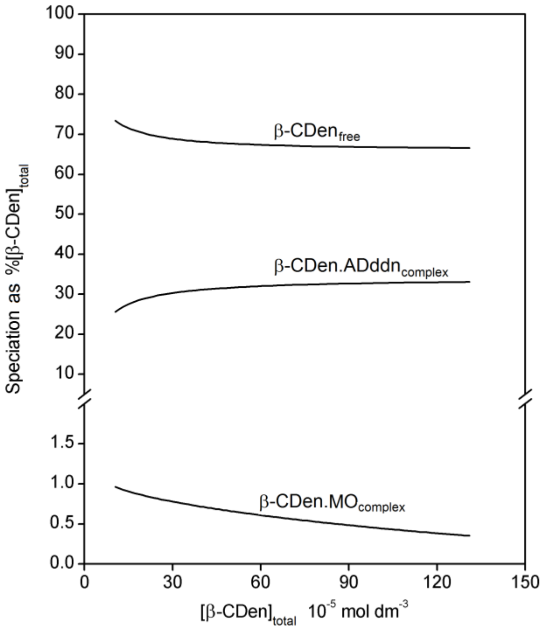

Figure S19: Speciation plot with [β-CDen]$_{total}$ = 100% for the PAAβ-CDen/PAAADddn/MO system.

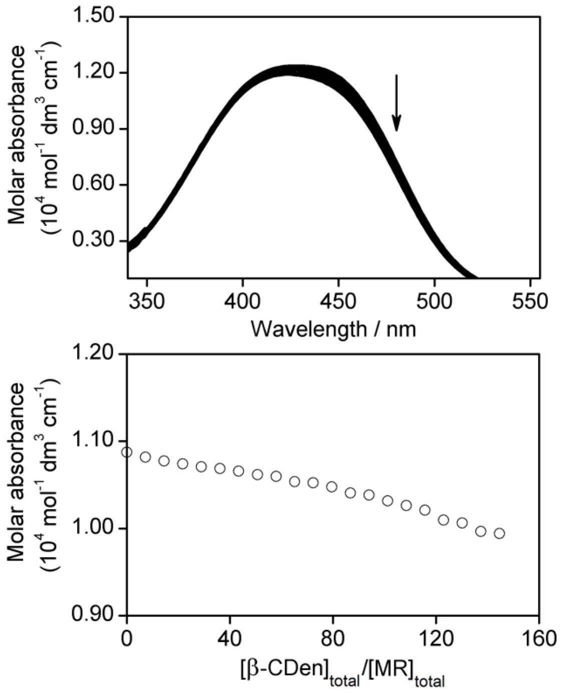

Figure S20: (Top) Molar absorbance variation of 1.5 cm^3 of a solution of MR ([MR] = 2.80×10^{-5} mol dm^{-3}) with 20 sequential additions of a PAAβ-CDen solution (25 mm^3 each, 2.59 wt %, [β-CDen] = 1.22×10^{-2} mol dm^{-3}) and a PAAADen solution (25 mm^3 each, 1.18 wt %, [ADen] = 4.03×10^{-3} mol dm^{-3}) at 298.2 K. All solutions were prepared in aqueous Na_2HPO_4/KH_2PO_4 buffer at pH 7.0 and I = 0.10 mol dm^{-3}. The arrow indicates the direction of molar absorbance variation with each addition of the PAAβ-CDen and PAAADen solutions. (Bottom) Molar absorbance variation at 460 nm is insufficient to fit to an algorithm for host-guest complexation of MR by β-CDen in the self-assembled PAAβ-CDen/PAAADen network.

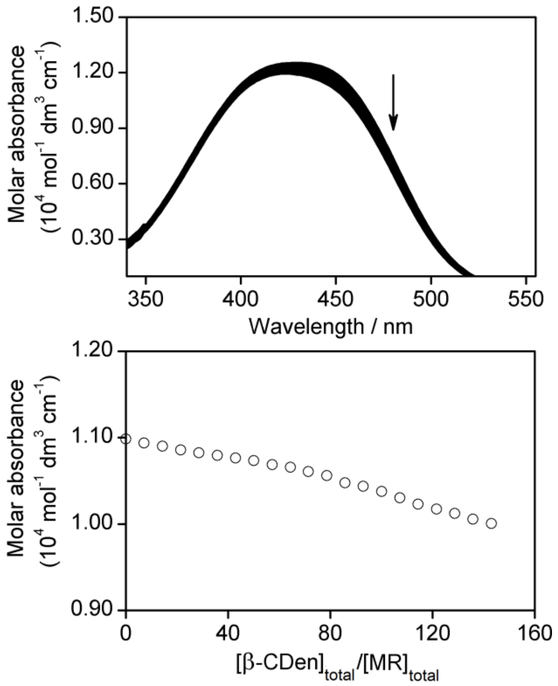

Figure S21: (Top) Molar absorbance variation 1.5 cm^3 of a solution of MR ([MR] = 2.80 × 10^{-5} mol dm^{-3}) with 20 sequential additions of a PAAβ-CDen solution (25 mm^3 each, 2.54 wt %, [β-CDen] = 1.20 × 10^{-2} mol dm^{-3}) and a PAAADhn solution (25 mm^3 each, 1.30 wt %, [ADhn] = 4.00 × 10^{-3} mol dm^{-3}) at 298.2 K. All solutions were prepared in aqueous Na_2HPO_4/KH_2PO_4 buffer at pH 7.0 and I = 0.10 mol dm^{-3}. The arrow indicates the direction of molar absorbance variation with each addition of the PAAβ-CDen and PAAADhn solutions. (Bottom) Molar absorbance variation at 460 nm is insufficient to fit to an algorithm for host-guest complexation of MR by β-CDen in the self-assembled PAAβ-CDen/PAAADhn network.

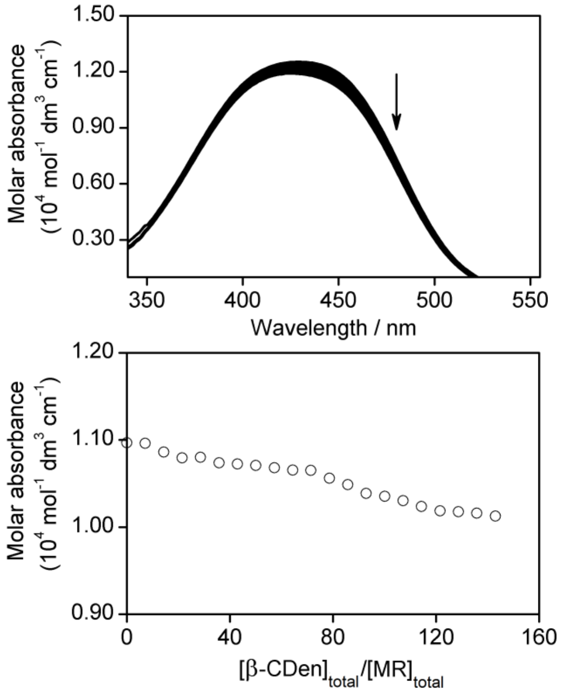

Figure S22: (Top) Molar absorbance variation of 1.5 cm^3 of a solution of MR ([MR] = 2.80 × 10^{-5} mol dm^{-3}) with 20 sequential additions of a PAAβ-CDen solution (25 mm^3 each, 2.54 wt %, [β-CDen] = 1.20 × 10^{-2} mol dm^{-3}) and a PAAADddn solution (25 mm^3 each, 1.37 wt %, [ADddn] = 4.01 × 10^{-3} mol dm^{-3}) at 298.2 K. All solutions were prepared in aqueous Na_2HPO_4/KH_2PO_4 buffer at pH 7.0 and I = 0.10 mol dm^{-3}. The arrow indicates the direction of molar absorbance variation with each addition of the PAAβ-CDen and PAAADddn solutions. (Bottom) Molar absorbance variation at 460 nm is insufficient to fit to an algorithm for host-guest complexation of MR by β-CDen in the self-assembled PAAβ-CDen/PAAADddn network.

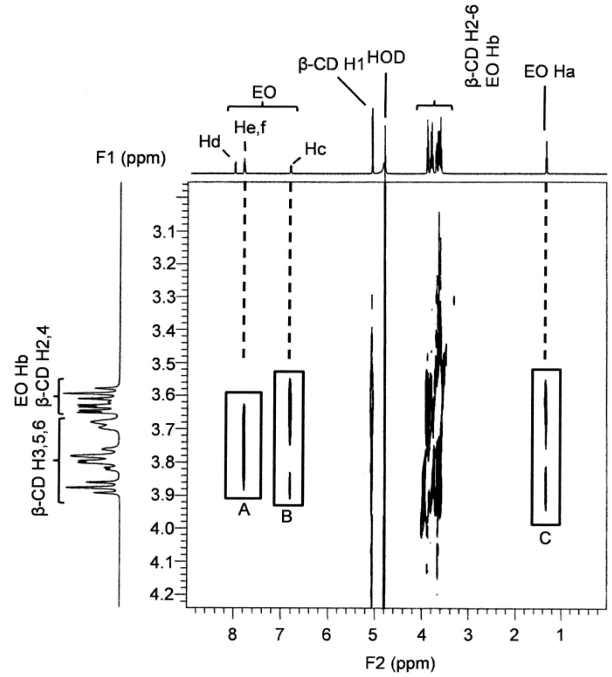

Figure S23: 2D ROESY ^{1}H NMR spectrum of EO ([EO] = 2.0 × 10^{-3} mol dm^{-3}) and β-CD ([β-CD] = 2.0 × 10^{-3} mol dm^{-3}) in D_2O Na_2HPO_4/KH_2PO_4 buffer solution at pD = 7.0 and I = 0.10 mol dm^{-3} at 298.2 K. Cross-peaks in boxes A, B, and C arise from dipolar interactions between the β-CD annular H3,5,6 protons and the EO aromatic (Hc, He and Hf) and methyl (Ha) protons, respectively. Dipolar interactions between EO methylene (Hb) protons and adjacent aromatic (Hc) and methyl (Ha) protons of EO, respectively, also contribute to the cross-peaks in boxes B and C, respectively.

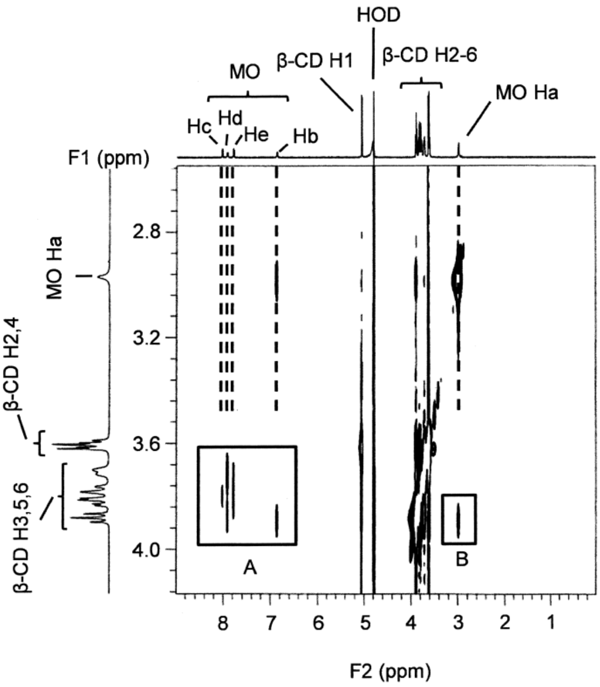

Figure S24: 2D ROESY ^{1}H NMR spectrum of MO ([MO] = 2.0 × 10^{-3} mol dm^{-3}) and β-CD ([β-CD] = 2.0 × 10^{-3} mol dm^{-3}) in D_2O Na_2HPO_4/KH_2PO_4 buffer solution at pD = 7.0 and I = 0.10 mol dm^{-3} at 298.2 K. Cross-peaks in boxes A and B arise from dipolar interactions between the β-CD annular H3,5,6 protons and the aromatic (Hb–e) and methyl (Ha) protons of MO, respectively.

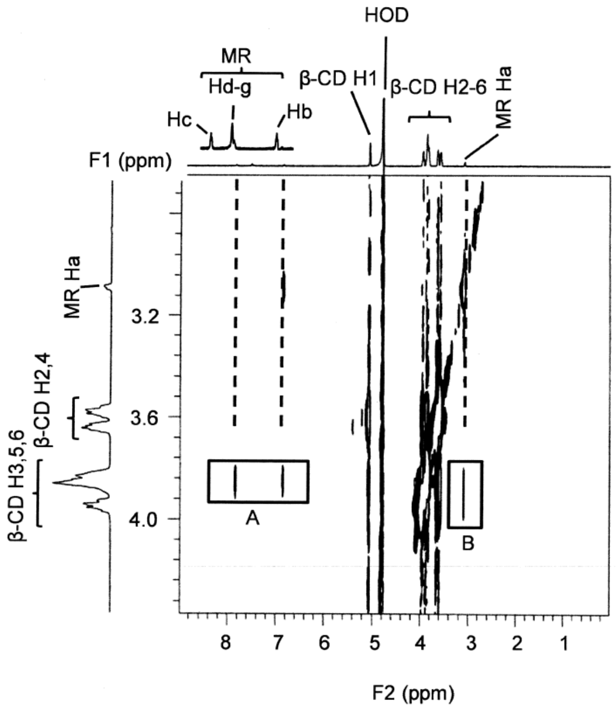

Figure S25: 2D ROESY ^{1}H NMR spectrum of MR ([MR] = 2.0 × 10^{-3} mol dm^{-3}) and β-CD ([β-CD] = 2.0 × 10^{-3} mol dm^{-3}) in D_2O Na_2HPO_4/KH_2PO_4 buffer solution at pD = 7.0 and I = 0.10 mol dm^{-3} at 298.2 K. Cross-peaks in boxes A and B arise from dipolar interactions between the β-CD annular H3,5,6 protons and the aromatic (Hb and Hc) and methyl (Ha) protons of MR, respectively.

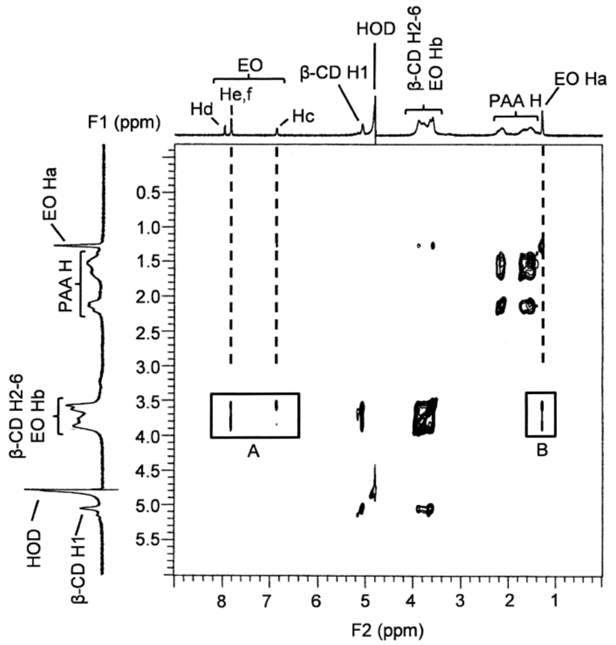

Figure S26: 2D NOESY ^{1}H NMR spectrum of EO ([EO] = 2.0 × 10^{-3} mol dm^{-3}) and PAAβ-CDen (0.44 wt %, [β-CDen] = 2.0 × 10^{-3} mol dm^{-3}) in D_2O Na_2HPO_4/KH_2PO_4 buffer solution at pD = 7.0 and I = 0.10 mol dm^{-3} at 298.2 K. Cross-peaks in boxes A and B arise from dipolar interactions between the annular H3,5,6 protons of β-CDen and the aromatic (Hc, He and Hf) and methyl (Ha) protons of EO, respectively.

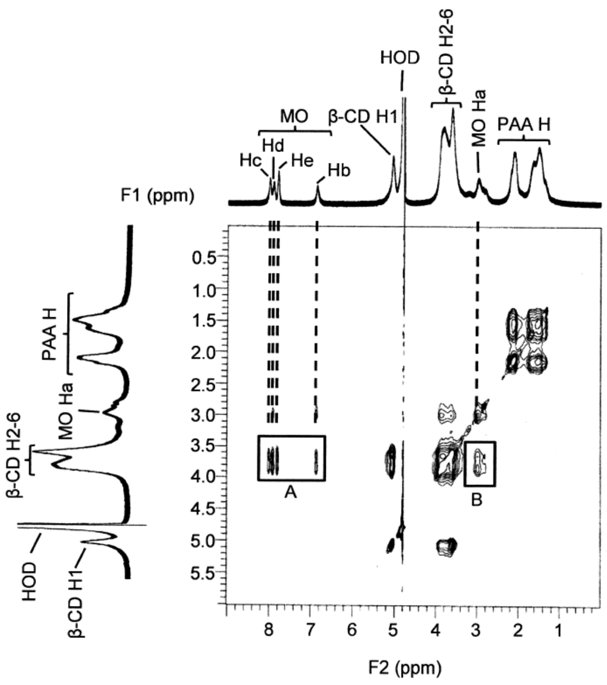

Figure S27: 2D NOESY ^{1}H NMR spectrum of MO ([MO] = 2.0 × 10^{-3} mol dm^{-3}) and PAAβ-CDen (0.44 wt %, [β-CDen] = 2.0 × 10^{-3} mol dm^{-3}) in D_2O Na_2HPO_4/KH_2PO_4 buffer solution at pD = 7.0 and I = 0.10 mol dm^{-3} at 298.2 K. Cross-peaks in boxes A and B arise from dipolar interactions between the annular H3,5,6 protons of β-CDen and the aromatic (Hb–e) and methyl (Ha) protons of MO, respectively.

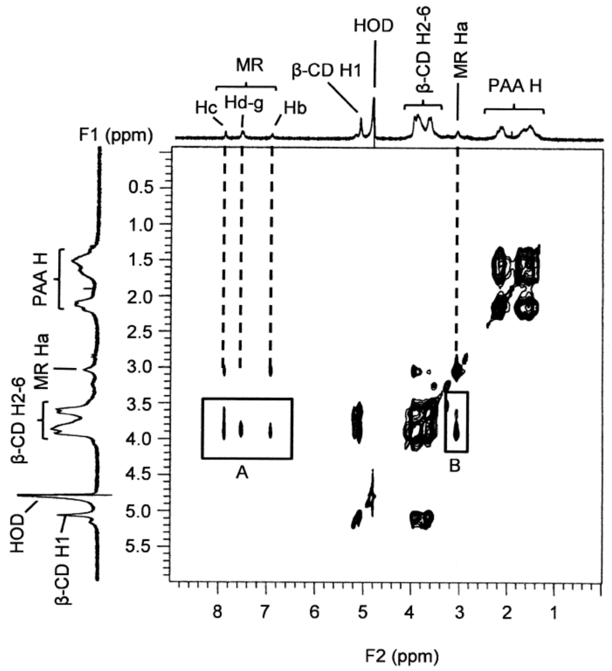

Figure S28: 2D NOESY ^{1}H NMR spectrum of MR ([MR] = 2.0 × 10^{-3} mol dm^{-3}) and PAAβ-CDen (0.44 wt %, [β-CDen] = 2.0 × 10^{-3} mol dm^{-3}) in D_2O Na_2HPO_4/KH_2PO_4 buffer solution at pD = 7.0 and I = 0.10 mol dm^{-3} at 298.2 K. Cross-peaks in boxes A and B arise from dipolar interactions between the annular H3,5,6 protons of β-CDen and the aromatic (Hb–g) and methyl (Ha) protons of MR, respectively.

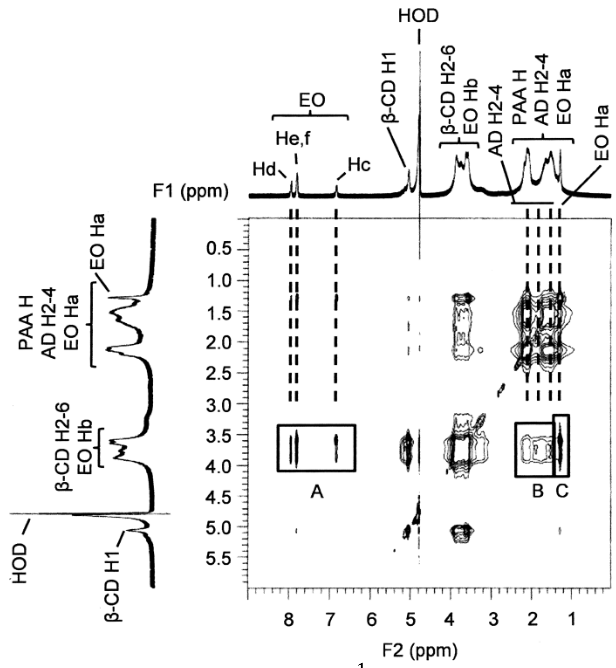

Figure S29: 2D NOESY ^{1}H NMR spectrum of EO ([EO] = 2.0 × 10^{-3} mol dm^{-3}) and PAAβ-CDen (0.78 wt %, [β-CDen] = 3.6 × 10^{-3} mol dm^{-3}) and PAAADen (0.36 wt %, [ADen] = 1.2 × 10^{-3} mol dm^{-3}) in D_2O Na_2HPO_4/KH_2PO_4 buffer solution at pD = 7.0 and I = 0.10 mol dm^{-3} at 298.2 K. Cross-peaks in boxes A and C arise from dipolar interactions between the annular H3,5,6 protons of β-CDen and the aromatic (Hc–f) and methyl (Ha) protons of EO, respectively. Cross-peaks in box B arise from dipolar interactions between the annular H3,5,6 protons of β-CDen and the H2–4 protons of ADen.

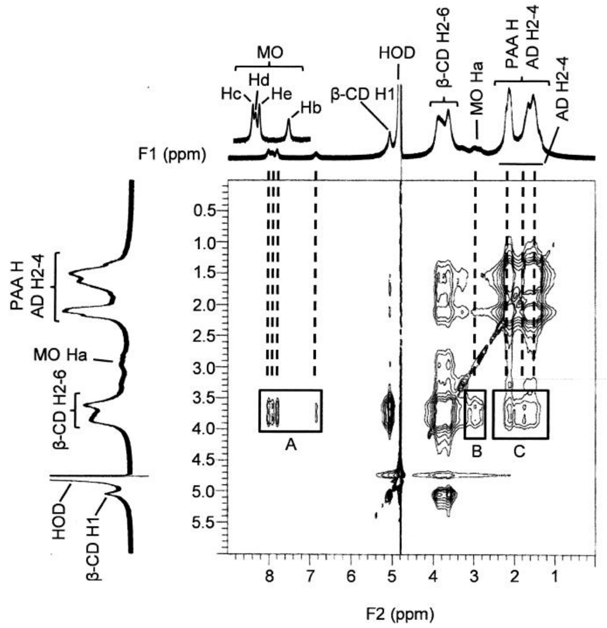

Figure S30: 2D NOESY ^{1}H NMR spectrum of MO ([MO] = 2.0 × 10^{-3} mol dm^{-3}) and PAAβ-CDen (0.78 wt %, [β-CDen] = 3.6 × 10^{-3} mol dm^{-3}) and PAAADen (0.36 wt %, [ADen] = 1.2 × 10^{-3} mol dm^{-3}) in D_2O Na_2HPO_4/KH_2PO_4 buffer solution at pD = 7.0 and I = 0.10 mol dm^{-3} at 298.2 K. Cross-peaks in boxes A and C arise from dipolar interactions between the annular H3,5,6 protons of β-CDen and the aromatic (Hb–e) and methyl (Ha) protons of MO, respectively. Cross-peaks in box B arise from dipolar interactions between the annular H3,5,6 protons of β-CDen and the H2–4 protons of ADen.

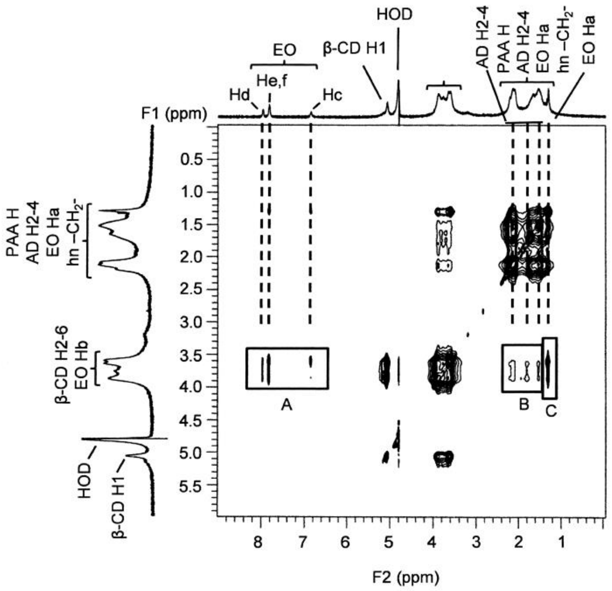

Figure S31: 2D NOESY ^{1}H NMR spectrum of EO ([EO] = 2.0 × 10^{-3} mol dm^{-3}) and PAAβ-CDen (0.78 wt %, [β-CDen] = 3.6 × 10^{-3} mol dm^{-3}) and PAAADhn (0.40 wt %, [ADhn] = 1.2 × 10^{-3} mol dm^{-3}) in D_2O Na_2HPO_4/KH_2PO_4 buffer solution at pD = 7.0 and I = 0.10 mol dm^{-3} at 298.2 K. Cross-peaks in boxes A and C arise from dipolar interactions between the annular H3,5,6 protons of β-CDen and the aromatic (Hc–f) and methyl (Ha) protons of EO, respectively. Cross-peaks in box B arise from dipolar interactions between the annular H3,5,6 protons of β-CD and the H2–4 protons of ADhn.

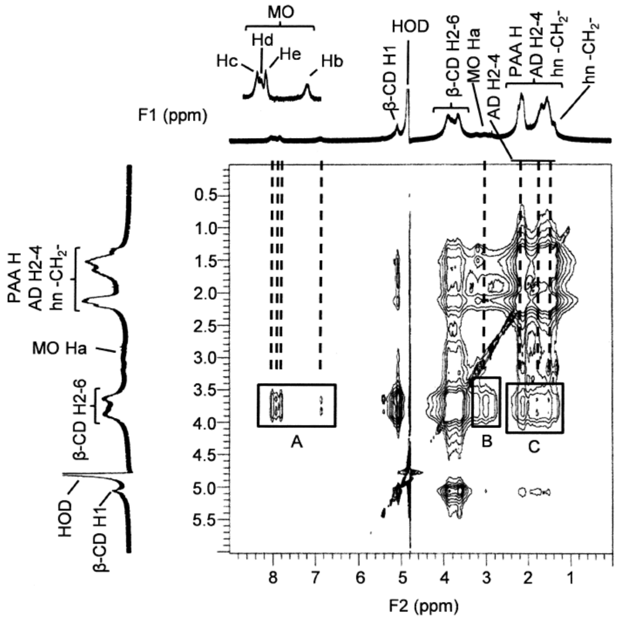

Figure S32: 2D NOESY ^{1}H NMR spectrum of MO ([MO] = 2.0 × 10^{-3} mol dm^{-3}) and PAAβ-CDen (0.78 wt %, [β-CDen] = 3.6 × 10^{-3} mol dm^{-3}) and PAAADhn (0.40 wt %, [ADhn] = 1.2 × 10^{-3} mol dm^{-3}) in D_2O Na_2HPO_4/KH_2PO_4 buffer solution at pD = 7.0 and I = 0.10 mol dm^{-3} at 298.2 K. Cross-peaks in boxes A and B arise from dipolar interactions between the annular H3,5,6 protons of *β*-CDen and the aromatic (Hb–e) and methyl (Ha) protons of MO, respectively. Cross-peaks in box C arise from dipolar interactions between the annular H3,5,6 protons of β-CDen and the H2–4 protons of ADhn.

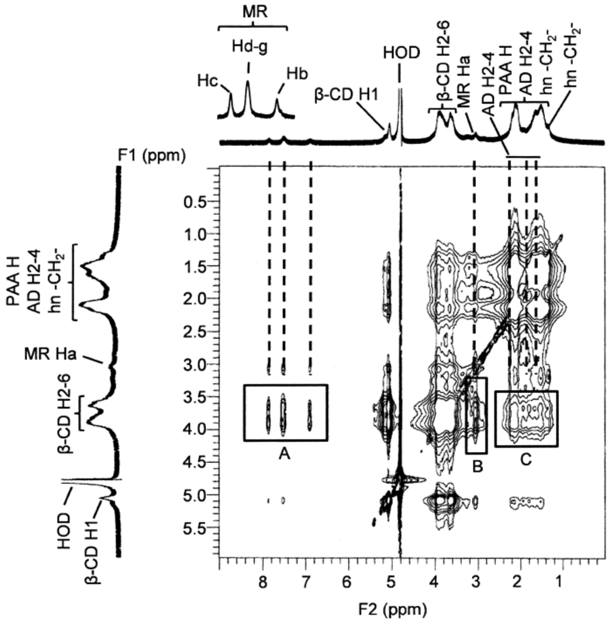

Figure S33: 2D NOESY ^{1}H NMR spectrum of MR ([MR] = 2.0 × 10^{-3} mol dm^{-3}) and PAAβ-CDen (0.78 wt %, [β-CDen] = 3.6 × 10^{-3} mol dm^{-3}) and PAAADhn (0.40 wt %, [ADhn] = 1.2 × 10^{-3} mol dm^{-3}) in D_2O Na_2HPO_4/KH_2PO_4 buffer solution at pD = 7.0 and I = 0.10 mol dm^{-3} at 298.2 K. Cross-peaks in boxes A and B arise from dipolar interactions between the annular H3,5,6 protons of [β-CDen and the aromatic (Hb–g) and methyl (Ha) protons of MR, respectively. Cross-peaks in box C arise from dipolar interactions between the annular H3,5,6 protons of [β-CDen and the H2–4 protons of ADhn.

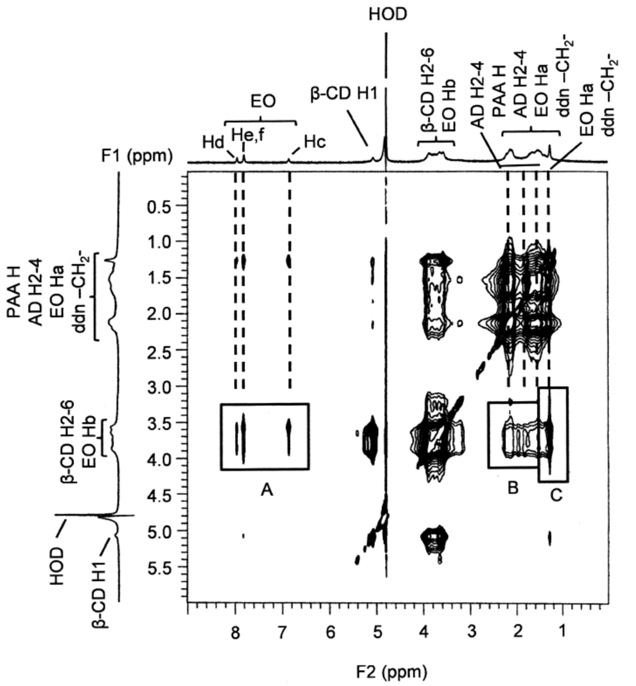

Figure S34: 2D NOESY ^{1}H NMR spectrum of EO ([EO] = 2.0 × 10^{-3} mol dm^{-3}) and PAAβ-CDen (0.78 wt %, [β-CDen] = 3.6 × 10^{-3} mol dm^{-3}) and PAAADddn (0.42 wt %, [ADddn] = 1.2 × 10^{-3} mol dm^{-3}) in D_2O Na_2HPO_4/KH_2PO_4 buffer solution at pD = 7.0 and I = 0.10 mol dm^{-3} at 298.2 K. Cross-peaks in box A arise from dipolar interactions between the annular H3,5,6 protons of β-CDen and the aromatic (Hc–f) protons of EO. Cross-peaks in box B arise from dipolar interactions between the annular H3,5,6 protons of β-CDen and the H2–4 protons of ADddn. Cross-peaks in box C arise from dipolar interactions between the annular H3,5,6 protons of β-CDen and the tether dodecyl methylene protons of ADddn and the methyl (Ha) protons of EO.

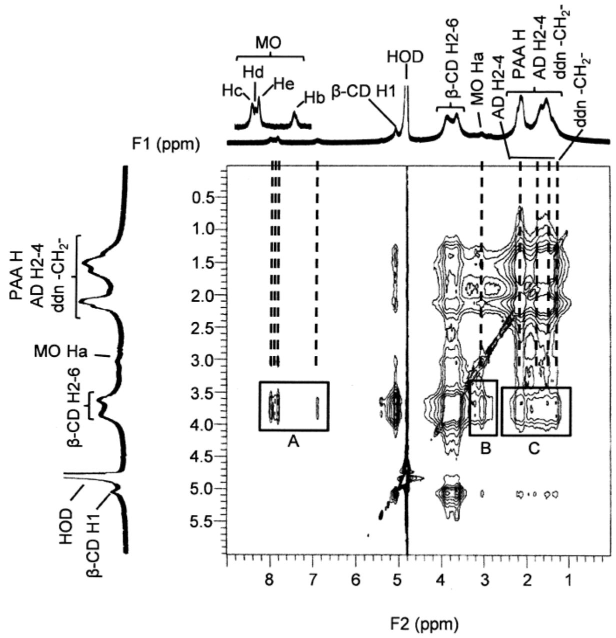

Figure S35: 2D NOESY ^{1}H NMR spectrum of MO ([MO] = 2.0 × 10^{-3} mol dm^{-3}) and PAAβ-CDen (0.78 wt %, [β-CDen] = 3.6 × 10^{-3} mol dm^{-3}) and PAAADddn (0.42 wt %, [ADddn] = 1.2 × 10^{-3} mol dm^{-3}) in D_2O Na_2HPO_4/KH_2PO_4 buffer solution at pD = 7.0 and I = 0.10 mol dm^{-3} at 298.2 K. Cross-peaks in boxes A and B arise from dipolar interactions between the annular H3,5,6 protons of β-CDen and the aromatic (Hb–e) and methyl (Ha) protons of MO, respectively. Cross-peaks in box C arise from dipolar interactions between the annular H3,5,6 protons of β-CDen and the H2–4 protons of the ADddn groups and the tether dodecyl methylene protons.

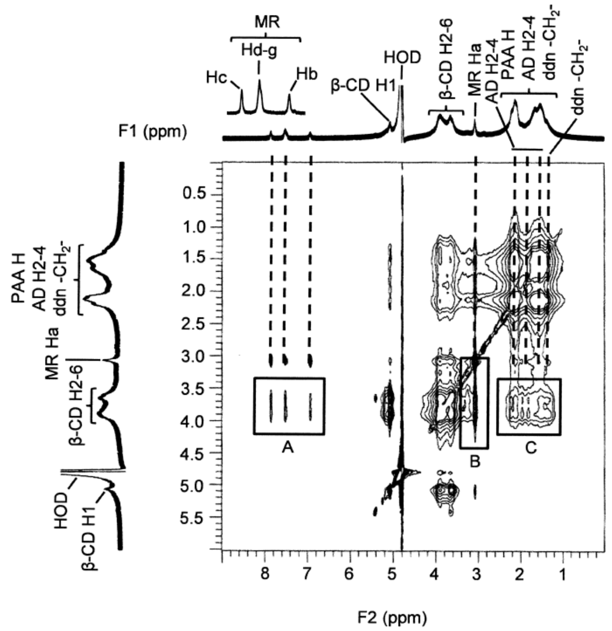

Figure S36: 2D NOESY ^{1}H NMR spectrum of MR ([MR] = 2.0 × 10^{-3} mol dm^{-3}) and PAAβ-CDen (0.78 wt %, [β-CDen] = 3.6 × 10^{-3} mol dm^{-3}) and PAAADddn (0.42 wt %, [ADddn] = 1.2 × 10^{-3} mol dm^{-3}) in D_2O Na_2HPO_4/KH_2PO_4 buffer solution at pD = 7.0 and I = 0.10 mol dm^{-3} at 298.2 K. Cross-peaks in boxes A and B arise from dipolar interactions between the annular H3,5,6 protons of β-CDen and the aromatic (Hb–g) and methyl (Ha) protons of MR, respectively. Cross-peaks in box C arise from dipolar interactions between the annular H3,5,6 protons of β-CDen and the H2–4 protons of ADddn and the tether dodecyl methylene protons.

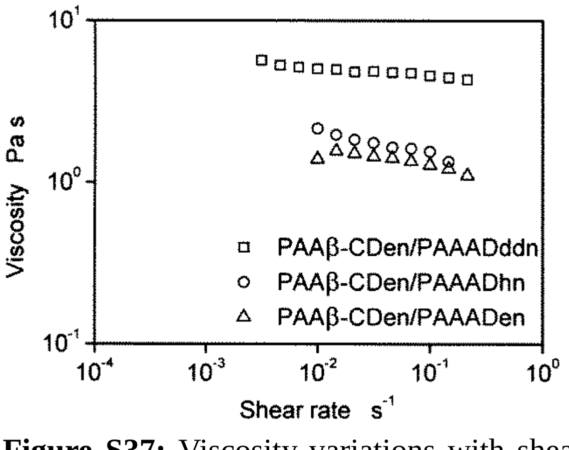

Figure S37: Viscosity variations with shear rate at 298.2 K of aqueous Na_2HPO_4/KH_2PO_4 buffered solutions (pH 7.0 and I = 0.10 mol dm^{-3}) of (g) 1.20 wt % PAAβ-CDen/PAAADddn: (0.78 wt %, [β-CDen] = 3.60 × 10^{-3} mol dm^{-3}) and PAAADddn (0.42 wt %, [ADddn] = 1.20 × 10^{-3} mol dm^{-3}), (h) 1.18 wt % PAAβ-CDen/PAAADhn: (0.78 wt %, [β-CDen] = 3.60 × 10^{-3} mol dm^{-3}) and PAAADhn (0.40 wt %, [ADhn] = 1.20 × 10^{-3} mol dm^{-3}) and (i) 1.14 wt % PAAβ-CDen/PAAADen: PAAβ-CDen (0.78 wt %, [β-CDen] = 3.60 × 10^{-3} mol dm^{-3}) and PAAADen (0.36 wt %, [ADen] = 1.20 × 10^{-3} mol dm^{-3}). These solutions correspond to (g), (h) and (i) in Table S1, but contain no dye.

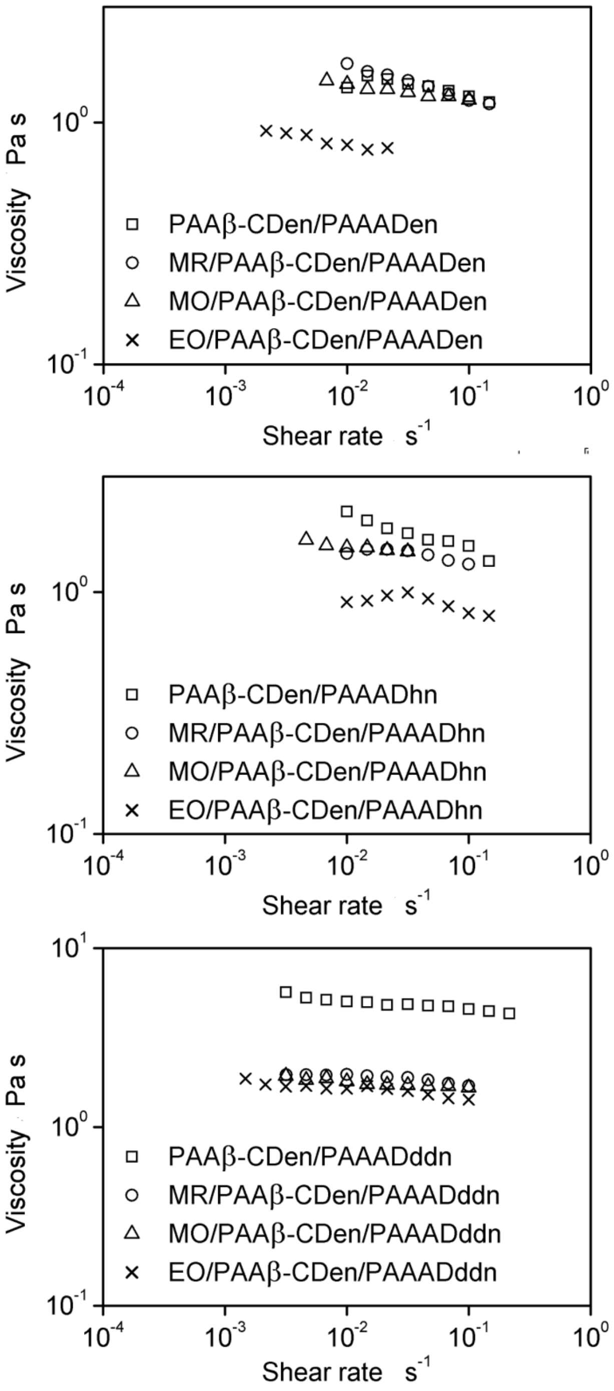

Figure S38: Viscosity variations with shear rate at 298.2 K of aqueous Na_2HPO_4/KH_2PO_4 buffered solutions (pH 7.0 and *I* = 0.10 mol dm^{-3}) of (Top) (i) 1.14 wt % PAAβ-CDen/PAAADen: PAAβ-CDen (0.78 wt %, [β-CDen] = 3.60 × 10^{-3} mol dm^{-3}) and PAAADen (0.36 wt %, [ADen] = 1.20 × 10^{-3} mol dm^{-3}), (Middle) (h) 1.18 wt % PAAβ-CDen/PAAADhn: (0.78 wt %, [β-CDen] = 3.60 × 10^{-3} mol dm^{-3}) and PAAADhn (0.40 wt %, [ADhn] = 1.20 × 10^{-3} mol dm^{-3}) and (Bottom) (g) 1.20 wt % PAAβ-CDen/PAAADddn: (0.78 wt %, [β-CDen] = 3.60 × 10^{-3} mol dm^{-3}) and PAAADddn (0.42 wt %, [ADddn] = 1.20 × 10^{-3} mol dm^{-3}) and 2.00 × 10^{-3} mol dm^{-3} MR, MO or EO. These solutions correspond to (i), (h) and (g) in Table S1.

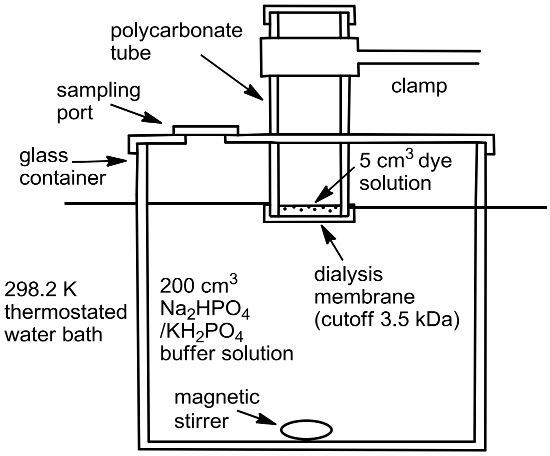

Figure S39: Schematic illustration of dye release measurement apparatus.

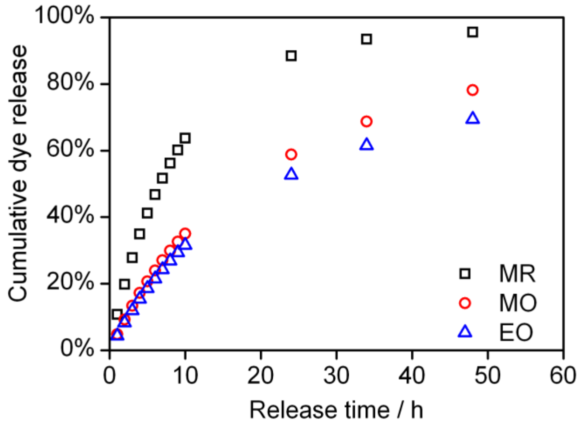

Figure S40: Release profiles of three dyes from a (j) 1.18 wt % PAA/PAAβ-CDen aqueous Na_2HPO_4/KH_2PO_4 buffer solution at pH 7.0 and I = 0.10 mol dm^{-3} at 298.2 K. The dye concentration = 2.00 × 10^{-3} mol dm^{-3}.

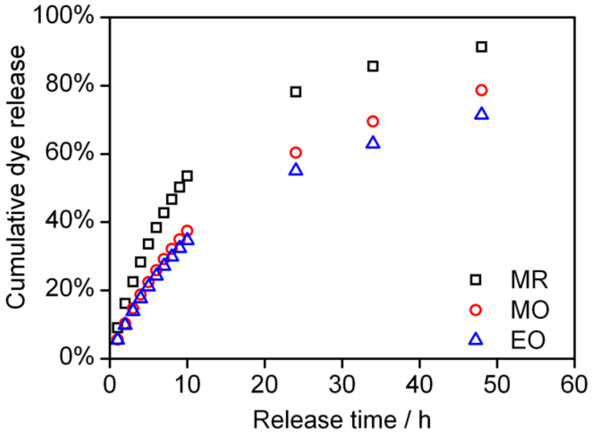

Figure S41: Release profiles of three dyes from a (i) 1.14 wt % PAAβ-CDen/PAAADen/dye Na_2HPO_4/KH_2PO_4 aqueous buffer solution at pH 7.0 and I = 0.10 mol dm^{-3} at 298.2 K. The initial dye concentration = 2.00 × 10^{-3} mol dm^{-3}

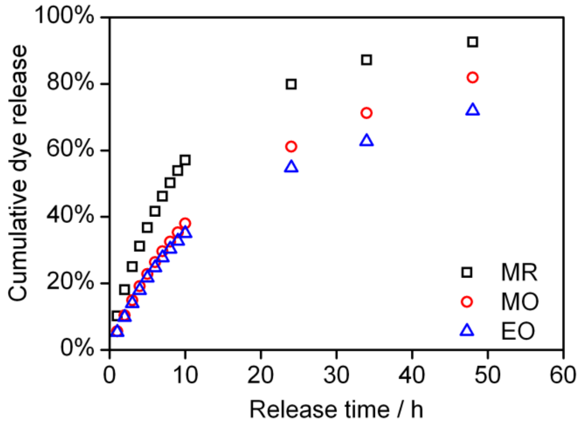

Figure S42: Release profiles of three dyes from a (h) 1.18 wt % PAAβ-CDen/PAAADhn aqueous Na_2HPO_4/KH_2PO_4 buffer solution at pH 7.0 and I = 0.10 mol dm^{-3} at 298.2 K. The dye concentration = 2.00 × 10^{-3} mol dm^{-3}.

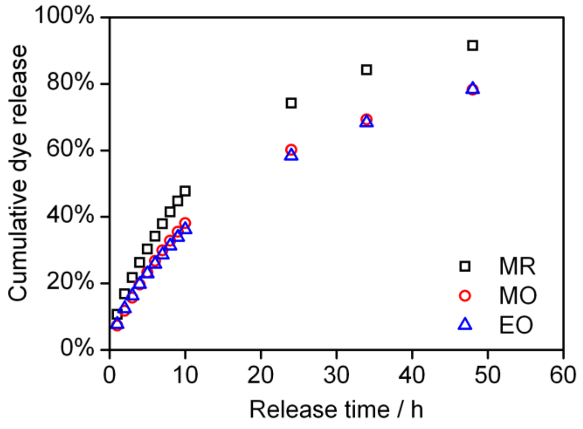

Figure S43: Release profiles of three dyes from a (g) 1.20 wt % PAAβ-CDen/PAAADddn aqueous Na_2HPO_4/KH_2PO_4 buffer solution at pH 7.0 and I = 0.10 mol dm^{-3} at 298.2 K. The dye concentration = 2.00 × 10^{-3} mol dm^{-3}.

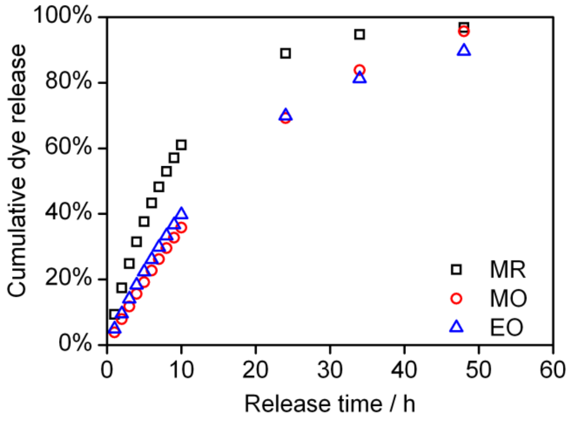

Figure S44: Release profiles of three dyes from a (f) 1.20 wt % PAA/β-CD aqueous Na_2HPO_4/KH_2PO_4 buffer solution at pH 7.0 and I = 0.10 mol dm^{-3} at 298.2 K. The dye concentration = 2.00 × 10^{-3} mol dm^{-3}.

Table S1: Composition of solutions for dye release studies.[1,2]

Solution		Composition
(a)	Na_2HPO_4/KH_2PO_4buffer	pH =7.0, I = 0.10 mol dm^{-3}
(b)	1.20 wt % PAA	PAA, 1.20 wt %
(e)	1.14 wt % PAA/PAAADen	PAAADen, 0.36 wt %,<br>[ADen substituents] = 1.20 × 10^{-3} mol dm^{-3}<br>PAA, 0.78 wt %, total wt % = 1.14
(d)	1.18 wt % PAA/PAAADhn	PAAADhn, 0.40 wt %,<br>[ADhn substituents] = 1.20 × 10^{-3} mol dm^{-3}<br>PAA, 0.78 wt %, total wt % = 1.18
(c)	1.20 wt % PAA/PAAADddn	PAAADddn, 0.42 wt %,<br>[ADddn substituents] = 1.20 × 10^{-3} mol dm^{-3}<br>PAA, 0.78 wt %, total wt % = 1.20
(f)	1.20 wt % PAA/β-CD	[β-CD] = 3.60 × 10^{-3} mol dm^{-3}<br>PAA, 1.20 wt %, total wt % = 1.20
(j)	1.18 wt % PAA/β-CDen	PAAβ-CDen, 0.78 wt %,<br>[β-CDen substituents] = 3.60 × 10^{-3} mol dm^{-3}<br>PAA, 0.40 wt %, total wt % = 1.18
(i)	1.14 wt % PAAβ-CDen/PAAADen	PAAβ-CDen, 0.78 wt %,<br>[β-CDen substituents] = 3.60 × 10^{-3} mol dm^{-3}<br>PAAADen, 0.36 wt %, total wt % = 1.14<br>[ADen substituents] = 1.20 × 10^{-3} mol dm^{-3}
(h)	1.18 wt % PAAβ-CDen/PAAADhn	PAAβ-CDen, 0.78 wt %,<br>[β-CDen substituents] = 3.60 × 10^{-3} mol dm^{-3}<br>PAAADhn, 0.40 wt %, total wt % = 1.18<br>[ADhn substituents] = 1.20 × 10^{-3} mol dm^{-3}
(g)	1.20 wt % PAAβ-CDen/PAAADddn	PAAβ-CDen, 0.78 wt %,<br>[β-CDen substituents] = 3.60 × 10^{-3} mol dm^{-3}<br>PAAADddn, 0.42 wt %, total wt % = 1.20<br>[ADddn substituents] = 1.20 × 10^{-3} mol dm^{-3}
(l)	1.89 wt % PAAβ-CDen/PAAADen	PAAβ-CDen, 1.30 wt %,<br>[β-CDen substituents] = 6.00 × 10^{-3} mol dm^{-3}<br>PAAADen, 0.59 wt %, total wt % =1.89<br>[ADen substituents] = 2.00 × 10^{-3} mol dm^{-3}
(k)	1.96 wt % PAAβ-CDen/PAAADhn	PAAβ-CDen, 1.30 wt %,<br>[β-CDen substituents] = 6.00 × 10^{-3} mol dm^{-3}<br>PAAADhn, 0.66 wt %, total wt % = 1.96<br>[ADhn substituents] = 2.00 × 10^{-3} mol dm^{-3}

[1]Solutions are labelled in the same order as in Figure 12 in the main manuscript.[2]All solutions were prepared in aqueous Na_2HPO_4/KH_2PO_4 buffer at pH 7.0 and I = 0.10 mol dm^{-3} at 298.2 K. Dye concentration was 2.00 × 10^{-3} mol dm^{-3} for all samples.
